# Of differing methods, disputed estimates and discordant interpretations: the meta-analytical multiverse of brain volume and IQ associations

**DOI:** 10.1098/rsos.211621

**Published:** 2022-05-11

**Authors:** Jakob Pietschnig, Daniel Gerdesmann, Michael Zeiler, Martin Voracek

**Affiliations:** ^1^ Department of Developmental and Educational Psychology, Faculty of Psychology, University of Vienna, Austria; ^2^ Department of Cognition, Emotion, and Methods in Psychology, Faculty of Psychology, University of Vienna, Austria; ^3^ Department of Physics Education, Faculty of Mathematics, Natural Sciences and Technology, University of Education Freiburg, Germany; ^4^ Department of Child and Adolescent Psychiatry, Medical University of Vienna, Austria

**Keywords:** *in vivo* brain volume, intelligence, meta-analysis, specification curve analysis, multiverse analysis, systematic review

## Abstract

Brain size and IQ are positively correlated. However, multiple meta-analyses have led to considerable differences in summary effect estimations, thus failing to provide a plausible effect estimate. Here we aim at resolving this issue by providing the largest meta-analysis and systematic review so far of the brain volume and IQ association (86 studies; 454 effect sizes from *k* = 194 independent samples; *N* = 26 000+) in three cognitive ability domains (full-scale, verbal, performance IQ). By means of competing meta-analytical approaches as well as combinatorial and specification curve analyses, we show that most reasonable estimates for the brain size and IQ link yield *r*-values in the mid-0.20s, with the most extreme specifications yielding *r*s of 0.10 and 0.37. Summary effects appeared to be somewhat inflated due to selective reporting, and cross-temporally decreasing effect sizes indicated a confounding decline effect, with three quarters of the summary effect estimations according to any reasonable specification not exceeding *r* = 0.26, thus contrasting effect sizes were observed in some prior related, but individual, meta-analytical specifications. Brain size and IQ associations yielded *r* = 0.24, with the strongest effects observed for more *g*-loaded tests and in healthy samples that generalize across participant sex and age bands.

## 1. Introduction

Associations between brain volume and intelligence have attracted the interest of the scientific community at least since the early 1800s [1]. This potential association is important for our understanding of (human) intelligence because brain volume is considered to be among the best-replicated correlates of psychometric *g* (i.e. general cognitive ability; [2]). In this vein, larger brains have been assumed to accommodate more neurons and glial cells which in turn may allow for more complex and quicker information processing. Other researchers have argued that larger brains provide surplus brain tissue that serves as a type of brain reserve, which may protect against a deterioration of cognitive abilities due to ageing- or fitness-related factors (for an overview, see [3]). However, to determine the potential relevance of these mechanisms, it is important to obtain a plausible estimate of the brain volume and IQ association.

Early empirical studies had to rely on proxies for intelligence (e.g. educational achievement) and brain size measures (such as head height, width, circumference or composites of it; e.g. [4]), which introduced considerable statistical noise in the data, thus leading to imprecise estimates of associations. Even with the advent of the first psychometric tests in the early 1900s, the issue of reliable brain volume measurement pertained, thus failing to alleviate validity problems of the observed associations.

It was only the development of neuroimaging methods, such as magnetic resonance imaging (MRI), that allowed reliable assessments of *in vivo* brain volumes and consequently their link with intelligence. While the direction of the seemingly positive brain size and intelligence link was frequently reproduced, the observed strength varied considerably. The first available report of MRI-assessed brain volume and IQ correlations in healthy men suggested that this association explained an impressive 25% of variance (*r* = 0.51; [5]), thus representing a large effect according to the well-established effect size classification of Cohen [6]. However, subsequent replications appeared to yield predominantly effects in the small-to-moderate range.

Several narrative reviews have been published that aimed at clarifying the brain-volume and IQ link [7–12]. Although all of them concluded that there is overwhelming evidence for a positive relationship, the strength and consequently meaning of the effect remained unclear. However, this is unsurprising because narrative reviews (i) lack formalized procedures that would allow systematic syntheses of effect sizes or influences of potential moderators and (ii) are vulnerable to various forms of bias (e.g. [13], pp. 301–302).

Systematic numerical effect syntheses by means of meta-analytical approaches are often considered to represent a means to provide definite conclusions about a given research question (i.e. assuming that they have been adequately performed) because they can address both moderator effects and estimate potentially confounding dissemination biases. However, meta-analytic researchers that investigate an identical research question may arrive at surprisingly different conclusions.

It has been demonstrated that design and analytical choices as well as researcher degrees of freedom (decisions that are being made that affect e.g. the specification of inclusion criteria or the selected statistical approach) may lead to substantially varying results from meta-analyses although they are based on similar databases and examine identical research questions (see the multiverse and specification-curve approach to meta-analysis introduced by [14], adopting analogous approaches for primary data analysis, i.e. multiverse analysis, by [15], and specification-curve-analysis, by [16,17]). Typically, there are a variety of reasonable choices that need to be made in regard to any type of study (for an overview, see [18]) which different researchers may (dis)agree about. For meta-analyses in this context, it matters most *which* studies are included and *how* they are analysed [14].

Researchers typically have conceptual or methodological reasons to prefer certain specifications over others. However, other researchers may apply different but equally reasonable selection criteria and analysis approaches which necessarily will lead to different summary effect estimations. One prominent example of the effects that such differing methodological choices can have is the widely received but heavily criticized paper that seemingly showed larger death-tolls of hurricanes with female as opposed to those with male names [19] in the prestigious journal *PNAS*. Although the methodological choices of this study had been well-justified, they represented an extreme specification which represented a single possibility out of a total of 1728 reasonable specifications (i.e. based on a number of published opinions about how these data should have been analyzed) to analyze these data (i.e. under the assumption that all possible meaningful kinds of data to analyze and how to do it had been identified in this study), only 37 of which (i.e. 2.1%) would have led to significantly deadlier hurricanes with female names [16,17].

Such observations have led researchers to argue that data analyses in general and research syntheses in particular should not only rely on a single reasonable specification which may represent only one of many justifiable approaches to treat and analyze data. Providing a distribution of these reasonable approaches allows a multi-faceted evaluation of potential moderating variables in terms of how they affect an effect size in terms of accuracy and strength.

Naturally, researchers may be unaware of all potentially reasonable ways to analyze their data (i.e. both in terms of which data to analyze and how to do so) because they may be unaware about influential factors such as moderating variables. By means of a brute-force approach, all possible (but not necessarily reasonable) ways to calculate meta-analytical summary effects can inform a researcher about potentially meaningful specifications that she may have missed. This means that clustering of summary effects of certain data subsets within the framework of a combinatorial meta-analysis may allow an identification of previously unobserved moderators ([20]; for discussion and an application, [14]).

Moreover, the time-point a certain meta-analysis is performed at can affect its outcomes because of the so-called decline effect (i.e. effect sizes of early studies investigating a given research question are more often than not inflated, thus leading to systematic decreases of effect strengths over time; [21,22]).

So far three meta-analyses have been published about the association between brain size and IQ. They are an illustrative example of the influences on meta-analytical results in terms of which studies have been included as well as when and how a meta-analysis was performed. The first formal meta-analysis about *in vivo* brain size and IQ associations was published in 2005 [23], yielding evidence for a moderate effect of *r* = 0.33 (i.e. explaining about 11% of variance; *k* = 33 independent samples, *N* = 1530).

About a decade later, this account was updated in the wake of another meta-analysis [24] which included an extension of the search strategy to further literature databases and the revision of the inclusion criteria (i.e. extending the inclusion from associations of only healthy to patient-based samples as well). This investigation yielded a considerably lower summary effect, indicating a small-to-moderate association between brain volume and IQ of *r* = 0.24 (i.e. explaining about 6% of variance; *k* = 120 independent samples, *N* = 6778). Moreover, an application of several dissemination bias assessment methods indicated that this summary effect was likely an inflated estimate due to confounding dissemination bias.

Finally, in another meta-analysis [25], a subset of study effects that had been reported in Pietschnig *et al*. [24] was synthesized anew. This reanalysis of a selection of studies, focusing merely on a part of the extant research evidence (i.e. about a fifth of the available independent effect sizes, representing a mere fifth of the evidence that was available in 2015), yielded a moderate effect of *r* = 0.31 (explaining about 10% of variance; *k* = 32 independent samples, *N* = 1758), although the authors concluded that the true association with highly *g*-loaded tests (i.e. *g*-loadings indicate how close a given test is related with the general factor of intelligence; henceforth referred to as: *g*-ness) should amount to about *r* = 0.40 (i.e. corresponding to about 16% of explained variance). Importantly, the analysis of Gignac & Bates [25] represents a comparatively small data subset based on one particular (out of many reasonable) specifications from the data of Pietschnig *et al*. [24].

These inconsistent results of the meta-analytical effect estimates illustrate how different procedural choices in conducting a meta-analysis are. In fact, the inconsistencies between results of these very three meta-analyses have recently been highlighted as a textbook example of specification-dependent outcomes [14]. The considerable differences of the observed summary effects have obviously meaningful implications for the meaning of the brain volume and IQ link. For instance, when assuming 16% of explained variance of the brain volume and IQ association (i.e. corresponding to the interpretation of [25]), this would mean that 2.4 IQ points of this difference are merely due to their difference in brain size, when observing two individuals with a 10 IQ point difference. But when assuming 6% of explained variance (i.e. corresponding to the estimate of [24]), only 0.9 IQ points are explained by this correlation.

Obviously, such inconsistencies in effect estimations are undesirable. Although brain volume and IQ have consistently been shown to be positively linked in prior research syntheses, the strength and therefore meaningfulness of this link remains unresolved.

In the present case, these differing estimates may be predominantly attributed to two components. On the one hand, a larger estimate of the first published meta-analysis [23] compared to subsequent updates could be attributed to the decline effect, because published primary study effects and consequently meta-analytical summary effects tend to decrease over time [22]. However, a decline effect can be ruled out as the sole cause for these differences because the most recent meta-analysis about this topic [25] yielded a larger effect than the previous estimate. On the other hand, these inconsistencies may be attributable to the different (reasonable) choices that researchers make when conceptualizing their study (or, in certain cases, analysing their data).

In these past three meta-analyses, several differences in terms of such choices can be identified. For instance, the inclusion criteria differed considerably in terms of sample characteristics (healthy versus patients; children/adolescents versus adults). Similarly, the analyses differed in terms of their methodological approaches. While two meta-analyses [23,25] used the so-called psychometric approach as developed by Hunter & Schmidt (e.g. [26]), the third one used the approach as introduced by Hedges & Olkin [27]. These two approaches most notably differ in terms of their underlying philosophy. While the former focuses on potential summary effect underestimations due to suboptimal sampling and measurement inaccuracies in the primary studies, the latter focuses on effect inflation and confounding bias. Consequently, in the former approach larger summary effects are typically obtained because individual study effects are corrected for artefacts such as range restriction or unreliability prior to the effect synthesis. The latter approach yields smaller summary effects because uncorrected estimates are synthesized.

It is evident that these differences in *how* to synthesize *which* primary study data necessarily lead to differing results. However, evidently any individual choice of a certain way to synthesize the available data may be criticized in its own right, even if this choice has been reasonably justified, thus leaving the meaning of contradictory findings of isolated meta-analytical summary estimates unresolved.

Novel methods provide a means to explore the multiverse of different design choices by allowing the assessment of a large number of (reasonable) specifications for the effect size synthesis in any given research question, thus providing a range of plausible estimates instead of an isolated point estimate (see [14]).

In the present preregistered meta-analysis of the associations between *in vivo* brain volume and cognitive abilities (intelligence, IQ), we aimed at resolving the ambiguity of available effect estimates by (i) updating the available meta-analytical data base, (ii) assessing subgroup analysis- and meta-regression-based influences of moderators, (iii) investigating evidence of dissemination bias and (iv) providing a range of effect estimates based on a large number of (reasonable) effect syntheses based on evidence from combinatorial, multiverse analysis, and specification-curve approaches to meta-analysis (see [28], for a similarly designed research synthesis).

## 2. Methods

The present study was preregistered at the Open Science Framework (OSF; https://osf.io/r6gnk). Study materials, R-codes, and all data are available at https://osf.io/y6msp/.

### 2.1. Inclusion and exclusion criteria

In order to be included in the present meta-analysis the primary studies were required to fulfil four criteria. First, they had to assess the association between *in vivo* brain volume and IQ. Second, *in vivo* brain volume has had to be measured by either MRI or CT. Studies had to provide measurements of the whole brain volume (TBV or ICV). If both were reported, TBV was preferred over ICV. Associations with partial brain area volume were excluded. Third, intelligence had to be measured with psychometric intelligence tests. Fourth, in cases of data dependencies, studies with the most comprehensive account of parameters were preferred. If no such hierarchy could be established, the earliest published account was coded.

Effect sizes were coded separately according to intelligence domain (full-scale, verbal and performance IQ). We used two different analysis methods when multiple subtest correlations within a certain domain were reported. First, in line with standard approaches, we selected those estimates that were judged to be conceptually more closely related to the respective domain (e.g. preferring verbal comprehension over working memory correlations for verbal IQ analyses). Second, we used robust variance estimations (RVE; [29]) to account for data dependencies while retaining the information of all coded information.

In cases where potentially eligible studies did not provide sufficient information to calculate effect sizes, corresponding study authors were contacted. When no response was received, the respective study was excluded (when effect sizes were reported to be non-significant in published papers, but no correlation coefficient was given and no response was received, coefficients were set to zero according to standard meta-analytical procedures; [30], pp. 408–409; in our study 30 out of 454 effect sizes).

### 2.2. Literature search

To update the so far most comprehensive available data set of IQ and brain size correlations [24], we first searched the online literature databases PubMed, ISI Web of Science, Scopus and Google Scholar using the string: (brain size AND intelligen*) OR (brain volume AND intelligen*) OR (brain size AND IQ) OR (brain volume AND IQ).

Second, we used a forward citation search of all three published meta-analyses on the subject [23–25]. Third, we conducted an extensive search for unpublished accounts in grey literature databases, search engines and repositories, sources dedicated to theses and dissertations, conference materials and registries for active studies. Finally, reference lists of all identified studies were handsearched for additional potentially eligible studies. Individual search strings and covered databases are available from https://osf.io/7z2u3/. Sample sex ratio, sample type (healthy versus patient samples), test instrument, publication year, mean sample age, participant numbers, effect size estimates and volumetric as well as IQ test standard deviations, number of corrections to the correlation coefficient (e.g. controlling for body height or mass), publication status (i.e. published versus grey literature versus personal communication), sample age (children/adolescents versus adults), brain volume measurement type (TBV or ICV), IQ domain and the assumed *g*-loadedness of the respectively used intelligence test (*g-*ness: fair versus good versus excellent; these categories correspond to the three rating criteria of [25], indicating a rank score based on included test number, dimensions and correlation with *g*; testing time was presently not used as an evaluation criterion; for details refer to [25]) were recorded independently by two researchers (D.G., M.Z.). A coding book containing data sheets and a coding manual explaining all variables and their categories is available at https://osf.io/fr8g7/. Inconsistencies were resolved by discussion with a third independent coder (J.P.). All searches were updated on April 14, 2021.

### 2.3. Synthesis methods

Meta-analytical calculations were performed in R by means of the metafor package [31].

#### 2.3.1. Hedges and Olkin meta-analysis

A random-effects meta-analysis in the tradition of Hedges & Olkin [27] was conducted based on independent effect sizes. Effect syntheses were calculated based on Fisher's *z-*transformed values to account for the skewed distributions of Pearson *r*s. For ease of interpretation, results were back-transformed into the *r*-metric prior to reporting. Some researchers have criticized this procedure because it may introduce a substantial upward bias (see, [26]). Therefore, we conducted a sensitivity analysis with correlations corrected for this negative bias [32].

Effect sizes were weighted according to study precision. Precision of the effect size estimates is illustrated by 95% confidence intervals (CI) based on the Knapp-Hartung adjustment [33,34]. In sensitivity analyses, we conducted leave-one-out analyses to evaluate influences of individual studies on the overall effect size and assessed potential influences of outliers following the approach of Viechtbauer & Cheung [35].

#### 2.3.2. Psychometric meta-analysis

Within the framework of psychometric meta-analyses, researchers aim at accounting for potential measurement inaccuracy and sampling errors. Following the specifications of two previous meta-analyses [23,25], we accounted for direct range restriction only (i.e. using the Case II formula of [36], to correct correlations and the approach of [37], to estimate their standard errors; see, [38]), because (i) reliabilities of MRI and IQ measures are typically high and (ii) reliabilities were infrequently reported [23,25]. Here, we used the *n*-weighted Hunter and Schmidt estimator (HS) to calculate effect syntheses.

#### 2.3.3. Robust variance estimation

By means of this approach, we were able to include more than one effect size per sample within a particular study and intelligence domain, following current recommendations [39]. Data dependencies within domain-specific analyses (i.e. full-scale versus verbal versus performance IQ) were modelled using robust variance estimation within meta-regressions, thus allowing for including a maximum amount of available information of primary studies (i.e. inclusion of multiple effect sizes from identical participants; this is reasonable because full-scale, verbal and performance IQ results are typically highly intercorrelated), while avoiding inappropriate effect size weighting due to dependent data [40].

We used a correlated-effects model because data dependence was primarily caused by correlations between participants' domain intelligence scores from different (sub)domains. We used *τ*^2^ (random-effects maximum likelihood) and *ω*^2^ by means of simplistic methods of moments estimators to estimate weights [41]. Fisher's *z*-transformed correlation coefficients were used for analyses.

### 2.4. Moderator analyses

#### 2.4.1. Subgroup analyses

Influences of categorical variables were examined in a series of mixed-effects subgroup analyses. We assessed group differences according to sample type (healthy versus clinical), age (children/adolescents versus adults), sex (men versus women) and *g-*ness. Furthermore, another two supplemental subgroup analyses were performed to (i) further corroborate results of bias analyses (i.e. published in a peer-reviewed journal versus obtained from grey literature or through personal communication) and (ii) assess possible influences of different brain volume measurement types (i.e. total brain volume versus intracranial volume).

#### 2.4.2. Meta-regressions

##### 2.4.2.1. Single regressions

Initially, a series of single regressions of effect sizes on study publication years was calculated for each IQ domain, to assess evidence for a potential decline effect. Differences of associations between intelligence domains were evaluated by means of a RVE-based meta-regression. For each RVE-regression, we ran one model with and one model without intercept to examine differences of coefficients compared to full-scale IQ and regression-based effect estimates for each domain, respectively.

##### 2.4.2.2. Multiple regressions

Subsequently, theory-guided hierarchical multiple precision-weighted mixed-effects meta-regressions were calculated for each domain. In three blocks we included (i) *g-*ness (only for full-scale IQ analyses; fair/good versus excellent), publication year, publication status; (ii) male ratio, mean sample age and (iii) study goal (i.e. assessment of IQ and brain volume association was the primary study goal versus not), number of included covariates in study. Model fit between blocks was compared by means of likelihood ratio tests. In supplemental analyses, we repeated these calculations only in studies that reported total brain volume (results omitted for brevity).

### 2.5. Dissemination bias

We used several dissemination bias detection methods to assess potential influences of summary effect-biasing artefacts. The use of multiple detection methods is sensible because different methods have been shown to not be equally sensitive to different bias scenarios and sources [42,43].

Results of bias analyses were based on published data only (excepting direct comparisons) and we focused on healthy samples (i.e. following the specifications of [25], and [23]), because bias may be expected to be stronger in neurotypical samples (i.e. because in non-neurotypical samples, expectations of certain effect strength observations may be lower and reporting of effect sizes therefore less dependent on conforming to preceding observations). Fisher's *r*-to-*z* transformed correlation coefficients were used (excepting *p*-value-based analyses) and analyses were carried out separately for full-scale, verbal and performance IQ.

First, we provide power-enhanced funnel plots (i.e. sunset plots, see [44]; using the R package metaviz, [45]) comprising estimates for the median power of all studies, necessary true effect estimates for a median power reduction to 33% or 66%, results of the test of excess significance (TES; observed random-effects summary effects were used for study power calculations; [46]), as well as the *R*-Index for expected replicability [47].

Second, we used Sterne & Egger's regression approach [48] by regressing effect sizes on the standard normal deviate effect size of the inverse standard error (*p*-values < 0.10 were considered indicative of bias). Third, the trim-and-fill method [49] was applied to detect potential funnel plot asymmetry. Resulting bias-adjusted estimates were interpreted in terms of a sensitivity analysis rather than a corrected summary effect estimate.

Fourth, we used three novel methods that are based on the distribution of published *p*-values and provide a means to estimate summary effect sizes and detect evidence for *p*-hacking and dissemination bias. The methods *p*-curve [50], *p*-uniform [51] and *p*-uniform* [52] are based on the idea that *p*-values follow a uniform distribution when the true investigated effect of any given research question is zero. In *p*-curve and *p*-uniform, only significant *p*-values are included in the analyses. This makes sense, because significant effects should have an identical publication probability. Observed right-skewness of these distributions are interpreted to be indicative of a true non-zero effect while observed left-skewness indicates confounding effects of *p*-hacking. Examinations of *p-*curves allow consequently a formal test of *p*-hacking by examining the shape of the observed *p*-value distribution (*p*-curve) or comparing observed with expected conditional *p*-values (*p*-uniform). By minimizing a loss function resulting from inspection of the observed *p*-value distribution (*p*-curve) or assessing the value for which the conditional *p*-value distribution is uniform (*p*-uniform), summary effects can be estimated.

Effect-size estimations that are based on *p*-uniform* rely on both significant and non-significant *p*-values (here, it is assumed that publication probabilities are identical within, but not across, groups). *P*-uniform has been shown to be more precise than both *p*-curve and *p*-uniform in the presence of non-trivial between-studies heterogeneity and allows assessment of the extent of unobserved heterogeneity [52]. *P*-uniform and *p*-uniform* were calculated by means of the R package puniform [53] and the R code available from www.p-curve.com/Supplement.

Fifth, we used two selection-model approaches that were either based on the four identical weight functions for *p*-values, as used in Pietschnig *et al*. [24]), or on the standard error of the effect sizes [54]. By means of these approaches, the robustness of the summary effect can be assessed when primary study effects are weighted according to them being more or less significant (*p*-values were weighted according to the approach of [55]) and more or less accurate [54]. We used metasens [56] to conduct the Copas and Shi analysis.

Sixth, we inspected the overlap between two DerSimonian-Laird estimator-based confidence intervals by means of the approach of Henmi & Copas [57]. This method can be seen as a type of sensitivity analysis in which conventionally calculated confidence intervals are compared with those of a hybrid estimation resulting from the use of fixed-effect weights but random-effects heterogeneity estimates. Large discrepancies between effect and confidence interval estimates are considered to be indicative of bias, although there is no consensus about a suitable hard-and-fast decision criterion.

Seventh, we directly assessed potential bias by regressing effect sizes on publication type (published versus unpublished effects). Moreover, we assessed cross-temporal changes in effect size in another meta-regression model, in order to assess the evidence for a potentially confounding decline effect in the data, as evidenced in a predecessor meta-analysis on this topic [24].

Finally, we conducted two cumulative meta-analyses according to (i) sample size and (ii) publication year to further illustrate potential influences of small-study effects and cross-temporal changes.

### 2.6. Exploring the multiverse

To untangle specific analytical design choices (i.e. reflecting researcher degrees of freedom) from the general pattern of brain size and IQ associations, we explored the multiverse of possible (reasonable) meta-analytic design specifications [14] using the R Code available from https://osf.io/kqgey/.

#### 2.6.1 Combinatorial meta-analysis

To this end, we first used a combinatorial meta-analysis to examine estimates for a large random selection of any possible subset of the 2*^k^* − 1 possible (not necessarily reasonable) combinations of the available data [20]. This approach can be interpreted as a sensitivity analysis by allowing the identification of outlier studies that overproportionally influence effect estimations.

Consequently, 2^123^ and 2^71^ (about 10 undecillions and 2 undecillions) combinations were possible for an exhaustive selection of full-scale IQ subsets for healthy and patient samples, respectively (2^70^ and 2^45^ as well as 2^49^ and 2^33^ combinations were possible for verbal and performance IQ). For our analyses, we randomly drew 100 000 subsets out of these possible combinations to illustrate outlier influences in GOSH plots (Graphic Display of Heterogeneity). Specifically, GOSH plots allow a visual inspection of summary effect distributions and their associated between-studies heterogeneity when any kind of (un)reasonable specification has been used. Moreover, numerical inspections of dispersion values (e.g. interquartile ranges) and the effect distribution permit an evaluation of the influence of moderating variables (i.e. narrow intervals and symmetrical distributions indicated well-interpretable summary effects).

#### 2.6.2 Specification-curve meta-analysis

By means of another method, we examined several possible reasonable specifications. In our meta-analytic specification-curve analyses (see [14]), we introduced three *which* factors: (1) sample age: adults versus children/adolescents versus either; (2) sample type: healthy versus patient versus either; (3) *g-*ness (for full-scale IQ only): fair or good versus excellent versus either; and two *how* factors: (1) effect size type: non-transformed Pearson *r*s versus *r*-to-*z*-transformed effect size versus small sample bias corrected *r*s versus range departure corrected *r*s; (2) analysis approach: Hedges-Olkin random-effects estimation versus Hunter-Schmidt effect estimation versus unweighted estimation versus RVE).

Consequently, these potentially influential analytic choices yielded 3 × 3 × 3 = 27 ways for which data to analyze and 4 × 4 = 16 ways for how to do it, thus totaling 27 × 16 = 432 reasonable specifications. In our further analyses in the subsets of verbal and performance data only, this number was reduced to 144 reasonable specifications because *g-*ness was not relevant in these cases. Specifications that comprised less than two effect sizes were dropped from analyses.

### 2.7. Final sample

**Figure 1.**
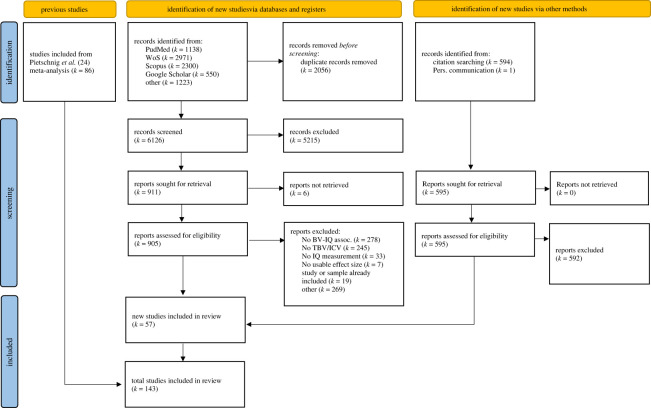
PRISMA flowchart.

We included effect sizes of 86 studies from Pietschnig *et al*. [24] ([58] and [59] were presently excluded because (i) of duplicate effect size publication and (ii) brain-volume measurements had not been performed *in vivo*) as well as 57 newly identified studies. Because more recent studies were based on larger samples, this update more than tripled the included participant numbers compared to Pietschnig *et al*. [24], thus representing a crucial expansion of the empirical knowledge base. In all, we extracted 454 effect sizes including 194 independent samples for full-scale, 115 for verbal, and 82 for performance IQ associations (*N*s = 26 764; 7667; and 5984; respectively). The majority of samples comprised healthy participants and about a third comprised patients (*k*s = 123 versus 71; 70 versus 45; 49 versus 33; *N*s = 23 403 versus 5361; 5440 versus 2237; 4162 versus 1858 for healthy and patient samples for full-scale, verbal and performance IQ, respectively). A PRISMA flowchart for the study inclusion process is provided in figure 1 (for study characteristics, table 1).

**Table 1.** Characteristics of included studies. Note. NA = info not available; Review: 1 = included in McDaniel [23], 2 = included in Pietschnig *et al*. [24], 3 = included in Gignac & Bates [25], 4 = included in present update; Reporting: reported = published in a journal article, grey = published as thesis/dissertation, PC = result obtained via personal communication; FSIQ = full-scale IQ; Type of test: IQ assessment used in study; subtest abbreviations: arith = arithmetic, bd = block design, com = comprehension, ds = digit symbol, inf = information, lm = logical memory, lns = letter-number sequencing, mr = matrix reasoning, obj = object assembly, pc = picture completion, pic = picture arrangement, sim = similarities, span = digit span (b stand for backwards), ss = symbol search, ss p + f = spatial span forwards and backwards, vpa = verbal pair associates; domain indices of the Wechsler scales are abbreviated as follows: POI = perceptual organization index, PRI = perceptual reasoning index, PSI = processing speed index, VCI = verbal comprehension index, WMI = working memory index; full information explaining all abbreviations are available in the codebook and data files in supplemental materials. Published study outcomes with *r* = exactly 0 represent correlations set to zero, because no eligible numerical value was available.

study	year	review	sample type	mean age	male ratio (%)	reporting	IQ domain	test	*n*	*r*
Yeo *et al*. [60]	1987	2, 4	patients	38.40	34.00	reported	FSIQ	WAIS	41	0.007
Yeo *et al*. [60]	1987	2, 4	patients	38.40	34.00	reported	verbal	WAIS verbal	41	0.12
Yeo *et al*. [60]	1987	2, 4	patients	38.40	34.00	reported	performance	WAIS performance	41	0.06
Willerman *et al*. [5]	1991	1, 2, 3, 4	healthy	18.90	0.00	reported	FSIQ	WAIS-R: voc, sim, bd, pc	20	0.33
Willerman *et al*. [5]	1991	1, 2, 3, 4	healthy	18.90	100.00	reported	FSIQ	WAIS-R: voc, sim, bd, pc	20	0.51
Andreasen *et al*. [61]	1993	1, 2, 3, 4	healthy	38.00	0.00	reported	FSIQ	WAIS-R	30	0.44
Andreasen *et al*. [61]	1993	1, 2, 3, 4	healthy	38.00	100.00	reported	FSIQ	WAIS-R	37	0.40
Andreasen *et al*. [61]	1993	2, 4	healthy	38.00	0.00	reported	verbal	WAIS-R verbal	30	0.43
Andreasen *et al*. [61]	1993	2,4	healthy	38.00	100.00	reported	verbal	WAIS-R verbal	37	0.33
Andreasen *et al*. [61]	1993	2, 4	healthy	38.00	0.00	reported	performance	WAIS-R performance	30	0.30
Andreasen *et al*. [61]	1993	2, 4	healthy	38.00	100.00	reported	performance	WAIS-R performance	37	0.43
Raz *et al*. [62]	1993	1, 2, 3, 4	healthy	43.80	59.00	reported	fluid	CFIT	29	0.43
Raz *et al*. [62]	1993	2, 4	healthy	43.80	59.00	reported	verbal	Extended Vocabulary (V3)	29	0.10
Castellanos *et al*. [63]	1994	1, 2, 4	healthy	12.10	100.00	reported	verbal	WISC-R: voc	46	0.33
Harvey *et al*. [64]	1994	2, 4	patients	35.60	38.00	reported	verbal	NART	26	0.38
Harvey *et al*. [64]	1994	2, 4	patients	31.10	77.00	reported	verbal	NART	48	0.24
Harvey *et al*. [64]	1994	2, 4	healthy	31.60	55.00	reported	verbal	NART	34	0.69
Jones *et al*. [65]	1994	2, 4	healthy	31.70	64.00	reported	verbal	NART or WAIS-R verbal	67	0.30
Wickett *et al*. [66]	1994	1, 2, 3, 4	healthy	25.00	0.00	reported	FSIQ	MAB	40	0.40
Wickett *et al*. [66]	1994	2, 4	healthy	25.00	0.00	reported	verbal	MAB verbal	40	0.44
Wickett *et al*. [66]	1994	2, 4	healthy	25.00	0.00	reported	performance	MAB performance	40	0.28
Bigler [67]	1995	2, 4	patients	29.40	71.00	reported	FSIQ	WAIS-R	72	−0.03
Egan *et al*. [68]	1995	1, 2, 3, 4	healthy	22.50	100.00	reported	FSIQ	WAIS-R	40	0.31
Egan *et al*. [68]	1995	2, 4	healthy	22.50	100.00	reported	verbal	WAIS-R verbal	40	0.21
Egan *et al*. [68]	1995	2, 4	healthy	22.50	100.00	reported	performance	WAIS-R performance	40	0.22
Haier *et al*. [69]	1995	2, 4	patients	26.39	54.00	reported	FSIQ	WAIS-R	28	0.65
Kareken *et al*. [70]	1995	1, 2, 4	healthy	27.66	63.00	PC	FSIQ	WAIS-R	68	0.30
Kareken *et al*. [70]	1995	4	patients	29.75	63.00	reported	verbal	COWA, Animal Naming, Boston Naming, Token Test, WRAT: Reading	68	0.36
Kareken *et al*. [70]	1995	4	healthy	27.66	63.00	reported	verbal	COWA, Animal Naming, Boston Naming, Token Test, WRAT: Reading	68	0.24
Kareken *et al*. [70]	1995	4	patients	29.75	63.00	reported	performance	WAIS-R: bd; Benton Line Orientation, Geometric Figure Drawings	68	0.18
Kareken *et al*. [70]	1995	4	healthy	27.66	63.00	reported	performance	WAIS-R: bd; Benton Line Orientation, Geometric Figure Drawings	68	0.26
Raz *et al*. [71]	1995	2, 4	patients	35.20	77.00	reported	FSIQ	WPPSI-R + BCS	11	−0.24
Reiss *et al*. [72]	1995	2, 4	healthy	11.28	42.00	PC	FSIQ	WISC-R or SB or BSID	87	0.00
Reiss *et al*. [72]	1995	2, 4	patients	10.80	35.00	reported	FSIQ	WISC-R or SB or BSID	51	0.25
Reiss *et al*. [73]	1996	1, 2, 4	healthy	10.60	0.00	PC	FSIQ	unknown FS	57	0.37
Reiss *et al*. [73]	1996	2, 4	healthy	10.10	100.00	PC	FSIQ	unknown FS	12	0.52
Blatter *et al*. [74]	1997	2, 4	patients	NA	NA	reported	verbal	WAIS-R verbal	22	0.57
Blatter *et al*. [74]	1997	2, 4	patients	NA	NA	reported	performance	WAIS-R performance	21	0.47
Mori *et al*. [75]	1997	2, 4	patients	70.20	38.00	reported	FSIQ	WAIS-R	60	0.40
Mori *et al*. [75]	1997	2, 4	patients	70.20	38.00	reported	verbal	WAIS-R verbal	60	0.37
Mori *et al*. [75]	1997	2, 4	patients	70.20	38.00	reported	performance	WAIS-R performance	60	0.37
Paradiso *et al*. [76]	1997	2, 3, 4	healthy	24.80	53.00	reported	FSIQ	WAIS-R	62	0.38
Paradiso *et al*. [76]	1997	2, 4	healthy	24.80	53.00	reported	verbal	WAIS-R: voc	62	0.27
Paradiso *et al*. [76]	1997	2, 4	healthy	24.80	53.00	reported	performance	WAIS-R: bd	62	0.32
Paradiso *et al*. [76]	1997	4	healthy	24.80	53.00	reported	verbal	WAIS-R: span	62	0.11
Flashman *et al*. [77]	1998	1, 2, 4	healthy	27.00	53.00	reported	FSIQ	WAIS-R	90	0.25
Flashman *et al*. [77]	1998	2, 4	healthy	27.00	53.00	reported	verbal	WAIS-R verbal	90	0.16
Flashman *et al*. [77]	1998	2, 4	healthy	27.00	53.00	reported	performance	WAIS-R performance	90	0.26
Gur *et al*. [78]	1999	1, 2, 3, 4	healthy	25.00	0.00	reported	FSIQ	WAIS-R: voc, bd; CVLT, JLO	40	0.40
Gur *et al*. [78]	1999	1, 2, 3, 4	healthy	27.00	100.00	reported	FSIQ	WAIS-R: voc, bd; CVLT, JLO	40	0.39
Gur *et al*. [78]	1999	2, 4	healthy	25.00	0.00	reported	verbal	WAIS-R: voc; CVLT	40	0.40
Gur *et al*. [78]	1999	2, 4	healthy	27.00	100.00	PC	verbal	WAIS-R: voc; CVLT	40	0.00
Gur *et al*. [78]	1999	4	healthy	25.00	0.00	reported	performance	WAIS-R: bd; JLO	40	0.57
Gur *et al*. [78]	1999	4	healthy	27.00	100.00	reported	performance	WAIS-R: bd; JLO	40	0.35
Leonard *et al*. [79]	1999	2, 4	patients	43.00	100.00	PC	verbal	WAIS-R verbal	37	0.00
Leonard *et al*. [79]	1999	2, 4	healthy	42.00	100.00	PC	verbal	WAIS-R verbal	33	0.00
Leonard *et al*. [79]	1999	2, 4	patients	43.00	100.00	PC	performance	WAIS-R performance	37	0.00
Leonard *et al*. [79]	1999	2, 4	healthy	42.00	100.00	PC	performance	WAIS-R performance	33	0.00
Tan *et al*. [80]	1999	1, 2, 3, 4	healthy	22.00	0.00	reported	fluid	CFIT	54	0.62
Tan *et al*. [80]	1999	1, 2, 3, 4	healthy	22.00	100.00	reported	fluid	CFIT	49	0.28
Warwick *et al*. [81]	1999	2, 4	patients	21.60	0.00	PC	verbal	Quick IQ Test [82]	11	0.00
Warwick *et al*. [81]	1999	2, 4	healthy	21.50	0.00	PC	verbal	Quick IQ Test [82]	13	0.00
Warwick *et al*. [81]	1999	2, 4	patients	21.80	100.00	PC	verbal	Quick IQ Test [82]	10	0.00
Warwick *et al*. [81]	1999	2, 4	patients	21.80	100.00	PC	verbal	Quick IQ Test [82]	10	0.00
Warwick *et al*. [81]	1999	2, 4	healthy	21.50	100.00	PC	verbal	Quick IQ Test [82]	25	0.00
Warwick *et al*. [81]	1999	2, 4	patients	21.63	100.00	reported	verbal	Quick IQ Test [82]	45	0.31
Warwick *et al*. [81]	1999	2, 4	patients	21.55	0.00	reported	verbal	Quick IQ Test [82]	24	0.53
Garde *et al*. [83]	2000	1, 2, 3, 4	healthy	80.70	0.00	PC	FSIQ	WAIS	22	0.22
Garde *et al*. [83]	2000	1, 2, 3, 4	healthy	80.70	100.00	PC	FSIQ	WAIS	46	0.07
Isaacs *et al*. [84]	2000	2, 4	healthy	7.75	73.00	PC	FSIQ	WISC-III	11	−0.03
Isaacs *et al*. [84]	2000	2, 4	healthy	7.75	38.00	PC	FSIQ	WISC-III	8	0.55
Isaacs *et al*. [84]	2000	2, 4	healthy	7.75	73.00	PC	verbal	WISC-III verbal	11	−0.04
Isaacs *et al*. [84]	2000	2, 4	healthy	7.75	38.00	PC	verbal	WISC-III verbal	8	0.57
Isaacs *et al*. [84]	2000	2, 4	healthy	7.75	73.00	PC	performance	WISC-III performance	11	−0.18
Isaacs *et al*. [84]	2000	2, 4	healthy	7.75	38.00	PC	performance	WISC-III performance	8	0.35
Kumra *et al*. [85]	2000	2, 4	patients	12.30	81.00	PC	FSIQ	WISC-III or WISC-R or WAIS: voc, bd	27	0.00
Kumra *et al*. [85]	2000	2, 4	patients	14.40	57.00	PC	FSIQ	WISC-III or WISC-R or WAIS: voc, bd	44	0.00
Lawson *et al*. [86]	2000	2, 4	patients	NA	NA	reported	FSIQ	WISC-III or WPPSI-R or DAS or SB or GMDS	47	0.43
Pennington *et al*. [87]	2000	1, 2, 4	healthy	19.06	44.00	reported	FSIQ	WISC-R or WAIS-R FS	36	0.31
Pennington *et al*. [87]	2000	2, 4	healthy	16.97	58.00	reported	FSIQ	WISC-R or WAIS-R FS	96	0.42
Schoenemann *et al*. [88]	2000	1, 2, 3, 4	healthy	23.20	0.00	PC	fluid	RSPM	72	0.21
Schoenemann *et al*. [88]	2000	2, 4	healthy	23.20	0.00	reported	verbal	MAB vocabulary	36	0.12
Wickett *et al*. [89]	2000	1, 2, 3, 4	healthy	24.97	100.00	reported	FSIQ	MAB	68	0.35
Wickett *et al*. [89]	2000	2, 4	healthy	24.97	100.00	reported	verbal	MAB verbal	68	0.33
Wickett *et al*. [89]	2000	2, 4	healthy	24.97	100.00	reported	performance	MAB performance	68	0.31
Castellanos *et al*. [90]	2001	2, 4	patients	9.70	0.00	reported	FSIQ	WISC-R or WISC-III: voc, bd	40	0.36
Coffey *et al*. [91]	2001	2, 4	healthy	74.85	38.00	reported	verbal	Verbal fluecy Task	319	−0.06
Coffey *et al*. [91]	2001	2, 4	healthy	74.85	38.00	reported	performance	WAIS-R: bd	318	0.06
Aylward *et al*. [92]	2002	1, 2, 4	healthy	NA	100.00	PC	FSIQ	unknown FS	46	−0.13
Aylward *et al*. [92]	2002	1, 2, 4	healthy	NA	NA	PC	FSIQ	unknown FS	30	0.08
Aylward *et al*. [92]	2002	2, 4	patients	18.80	87.00	reported	FSIQ	unknown FS	67	0.10
Aylward *et al*. [92]	2002	2, 4	patients	18.80	87.00	reported	verbal	unknown verbal	67	0.08
Aylward *et al*. [92]	2002	2, 4	healthy	18.90	92.00	reported	verbal	unknown verbal	83	−0.01
Aylward *et al*. [92]	2002	2, 4	patients	18.80	87.00	reported	performance	unknown performance	67	0.10
Aylward *et al*. [92]	2002	2, 4	healthy	18.90	92.00	reported	performance	unknown performance	83	0.09
MacLullich *et al*. [93]	2002	1, 2, 3, 4	healthy	67.80	100.00	reported	fluid	RSPM	95	0.39
MacLullich *et al*. [93]	2002	2, 4	healthy	67.80	100.00	reported	verbal	NART	97	0.30
Nosarti *et al*. [94]	2002	1, 2, 4	healthy	14.90	65.00	PC	FSIQ	unknown FS	42	0.37
Shapleske *et al*. [95]	2002	1, 2, 3, 4	healthy	33.30	100.00	PC	FSIQ	unknown FS	23	0.13
Collinson *et al*. [96]	2003	2, 4	healthy	16.40	60.00	PC	FSIQ	WISC-R or WAIS-R	22	−0.13
Collinson *et al*. [96]	2003	2, 4	patients	16.80	67.00	PC	FSIQ	WISC-R or WAIS-R	32	−0.27
Collinson *et al*. [96]	2003	2, 4	patients	16.80	67.00	PC	verbal	WISC-R or WAIS-R verbal	32	−0.28
Collinson *et al*. [96]	2003	2, 4	healthy	16.40	60.00	PC	verbal	WISC-R or WAIS-R verbal	22	−0.09
Collinson *et al*. [96]	2003	2, 4	patients	16.80	67.00	PC	performance	WISC-R or WAIS-R performance	32	−0.19
Collinson *et al*. [96]	2003	2, 4	healthy	16.40	60.00	PC	performance	WISC-R or WAIS-R performance	22	−0.17
Giedd [97]	2003	1, 2, 4	healthy	NA	0.00	PC	FSIQ	unknown FS	8	0.46
Giedd [97]	2003	1, 2, 4	healthy	NA	100.00	PC	FSIQ	unknown FS	7	0.17
Giedd [97]	2003	1, 2, 4	healthy	NA	0.00	PC	FSIQ	unknown FS	7	−0.67
Giedd [97]	2003	1, 2, 4	healthy	NA	100.00	PC	FSIQ	unknown FS	7	0.67
Giedd [97]	2003	1, 2, 4	healthy	NA	0.00	PC	FSIQ	unknown FS	39	0.34
Giedd [97]	2003	1, 2, 4	healthy	NA	100.00	PC	FSIQ	unknown FS	63	0.27
Kesler *et al*. [98]	2003	2, 4	patients	26.16	52.00	reported	FSIQ	WAIS-R	25	0.47
Kesler *et al*. [98]	2003	2, 4	patients	26.16	52.00	reported	verbal	WAIS-R verbal	25	0.57
Yurgelun-Todd *et al*. [99]	2003	2, 4	healthy	14.60	0.00	reported	FSIQ	Shipley total	24	0.20
Yurgelun-Todd *et al*. [99]	2003	2, 4	healthy	14.50	100.00	reported	FSIQ	Shipley total	13	0.26
Yurgelun-Todd *et al*. [99]	2003	2, 4	healthy	14.60	0.00	reported	verbal	Shipley verbal	24	0.17
Yurgelun-Todd *et al*. [99]	2003	2, 4	healthy	14.50	100.00	reported	verbal	Shipley verbal	13	0.19
Yurgelun-Todd *et al*. [99]	2003	4	healthy	14.60	0.00	reported	verbal	WAIS-III: span	24	0.19
Yurgelun-Todd *et al*. [99]	2003	4	healthy	14.50	100.00	reported	verbal	WAIS-III: span	13	0.55
Yurgelun-Todd *et al*. [99]	2003	4	healthy	14.60	0.00	reported	performance	WAIS-III: ds	24	0.07
Yurgelun-Todd *et al*. [99]	2003	4	healthy	14.50	100.00	reported	performance	WAIS-III: ds	13	0.48
Frangou *et al*. [100]	2004	1, 2, 4	healthy	15.05	50.00	reported	FSIQ	WISC-III or WAIS-III	40	0.41
Isaacs *et al*. [101]	2004	2, 4	healthy	15.90	0.00	PC	FSIQ	Wechsler FS	38	0.24
Isaacs *et al*. [101]	2004	2, 4	healthy	15.90	100.00	PC	FSIQ	Wechsler FS	38	0.27
Isaacs *et al*. [101]	2004	2, 4	healthy	14.86	50.00	PC	FSIQ	Wechsler FS	16	0.49
Isaacs *et al*. [101]	2004	2, 4	healthy	15.60	0.00	PC	verbal	Wechsler verbal	38	0.20
Isaacs *et al*. [101]	2004	2, 4	healthy	15.90	0.00	PC	performance	Wechsler performance	38	0.21
Isaacs *et al*. [101]	2004	2, 4	healthy	15.90	100.00	PC	performance	Wechsler performance	38	0.15
Ivanovic *et al*. [102,103]	2004	1, 2, 4	healthy	18.00	0.00	reported	FSIQ	WAIS-R	49	0.37
Ivanovic *et al*. [102,103]	2004	1, 2, 4	healthy	18.00	100.00	reported	FSIQ	WAIS-R	47	0.55
Ivanovic *et al*. [102,103]	2004	2, 4	healthy	18.00	0.00	reported	verbal	WAIS-R verbal	49	0.33
Ivanovic *et al*. [102,103]	2004	2, 4	healthy	18.00	100.00	reported	verbal	WAIS-R verbal	47	0.55
Ivanovic *et al*. [102,103]	2004	2, 4	healthy	18.00	0.00	reported	performance	WAIS-R performance	49	0.38
Ivanovic *et al*. [102,103]	2004	2, 4	healthy	18.00	100.00	reported	performance	WAIS-R performance	47	0.52
Rojas *et al*. [104]	2004	2, 3, 4	healthy	43.62	47.00	PC	FSIQ	WAIS-R or WAIS-III	17	0.31
Rojas *et al*. [104]	2004	2, 4	patients	30.30	87.00	PC	FSIQ	WAIS-R or WAIS-III	15	0.07
Rojas *et al*. [104]	2004	2, 4	patients	30.30	87.00	PC	verbal	WAIS-R or WAIS-III verbal	15	0.30
Rojas *et al*. [104]	2004	2, 4	healthy	43.62	47.00	PC	verbal	WAIS-R or WAIS-III verbal	17	0.19
Rojas *et al*. [104]	2004	2, 4	patients	30.30	87.00	PC	performance	WAIS-R or WAIS-III performance	15	0.15
Rojas *et al*. [104]	2004	2, 4	healthy	43.62	47.00	PC	performance	WAIS-R or WAIS-III performance	17	0.27
Toulopoulou *et al*. [105]	2004	2, 4	patients	42.23	50.00	reported	FSIQ	WAIS-R	201	0.28
Toulopoulou *et al*. [105]	2004	2, 4	patients	42.23	50.00	reported	verbal	WAIS-R verbal	201	0.28
Waiter *et al*. [106]	2004	2, 4	healthy	15.50	100.00	PC	FSIQ	WISC-III-R or WAIS-IV	16	0.13
Waiter *et al*. [106]	2004	2, 4	patients	15.40	100.00	PC	FSIQ	WISC-III-R or WAIS-IV	16	−0.06
Waiter *et al*. [106]	2004	2, 4	patients	15.40	100.00	PC	verbal	WISC-III-R or WAIS-IV verbal	16	−0.17
Waiter *et al*. [106]	2004	2, 4	healthy	15.50	100.00	PC	verbal	WISC-III-R or WAIS-IV verbal	16	0.20
Waiter *et al*. [106]	2004	2, 4	patients	15.40	100.00	PC	performance	WISC-III-R or WAIS-IV performance	16	0.10
Waiter *et al*. [106]	2004	2, 4	healthy	15.50	100.00	PC	performance	WISC-III-R or WAIS-IV performance	16	0.23
Antonova *et al*. [107]	2005	2, 4	patients	40.49	60.00	PC	verbal	WAIS-III: voc	44	0.16
Antonova *et al*. [107]	2005	2, 4	healthy	33.72	58.00	PC	verbal	WAIS-III: voc	43	0.24
Lodygensky *et al*. [108]	2005	2, 4	healthy	8.42	57.00	PC	FSIQ	WISC-R	21	0.46
Lodygensky *et al*. [108]	2005	2, 4	patients	8.58	53.00	PC	FSIQ	WISC-R	60	0.35
Thoma *et al*. [109]	2005	2, 3, 4	healthy	23.50	100.00	reported	FSIQ	RPM, TrailsAB, WAIS-R: voc, bd, ds; VMRT, COWA	19	0.27
Debbané *et al*. [110]	2006	2, 4	healthy	15.10	43.00	PC	FSIQ	WISC-III or WAIS-III	41	0.16
Debbané *et al*. [110]	2006	2, 4	patients	16.70	37.00	PC	FSIQ	WISC-III or WAIS-III	43	0.16
Rojas *et al*. [111]	2006	2, 4	healthy	21.41	100.00	PC	FSIQ	WAIS-III or WISC-III	23	0.46
Rojas *et al*. [111]	2006	2, 4	patients	20.79	100.00	PC	FSIQ	WAIS-III or WISC-III	24	0.30
Rojas *et al*. [111]	2006	2, 4	patients	20.79	100.00	PC	verbal	WAIS-III or WISC-III verbal	24	0.28
Rojas *et al*. [111]	2006	2, 4	healthy	21.41	100.00	PC	verbal	WAIS-III or WISC-III verbal	23	0.55
Rojas *et al*. [111]	2006	2, 4	patients	20.79	100.00	PC	performance	WAIS-III or WISC-III performance	24	0.31
Rojas *et al*. [111]	2006	2, 4	healthy	21.41	100.00	PC	performance	WAIS-III or WISC-III performance	23	0.09
Staff *et al*. [112]	2006	1, 2, 4	healthy	79.50	61.00	PC	fluid	RSPM	102	−0.10
Staff *et al*. [112]	2006	2, 4	healthy	79.50	61.00	PC	verbal	NART	102	−0.14
Voelbel *et al*. [113]	2006	2, 4	healthy	10.77	100.00	PC	FSIQ	WISC-III	13	−0.11
Voelbel *et al*. [113]	2006	2, 4	patients	10.16	100.00	PC	FSIQ	WISC-III	38	0.02
Voelbel *et al*. [113]	2006	2, 4	patients	10.08	100.00	PC	FSIQ	WISC-III	12	−0.14
Voelbel *et al*. [113]	2006	2, 4	patients	10.16	100.00	PC	verbal	WISC-III verbal	38	0.08
Voelbel *et al*. [113]	2006	2, 4	patients	10.08	100.00	PC	verbal	WISC-III verbal	12	0.23
Voelbel *et al*. [113]	2006	2, 4	healthy	10.77	100.00	PC	verbal	WISC-III verbal	13	−0.15
Voelbel *et al*. [113]	2006	2, 4	healthy	10.77	100.00	PC	performance	WISC-III performance	13	0.06
Voelbel *et al*. [113]	2006	2, 4	patients	10.08	100.00	PC	performance	WISC-III performance	12	−0.48
Voelbel *et al*. [113]	2006	2, 4	patients	10.16	100.00	PC	performance	WISC-III performance	38	−0.02
Wozniak *et al*. [114]	2006	2, 4	healthy	12.40	46.20	PC	FSIQ	WISC-III or WISC-IV	13	0.59
Wozniak *et al*. [114]	2006	2, 4	patients	12.30	50.00	PC	FSIQ	WISC-III or WISC-IV	14	0.41
Chiang *et al*. [115]	2007	2, 4	patients	29.20	45.00	reported	verbal	WAIS verbal	39	−0.02
Chiang *et al*. [115]	2007	2, 4	healthy	NA	NA	reported	verbal	WAIS verbal	16	−0.44
Chiang *et al*. [115]	2007	2, 4	patients	29.20	45.00	reported	performance	WAIS performance	39	0.10
Chiang *et al*. [115]	2007	2, 4	healthy	NA	NA	reported	performance	WAIS performance	16	0.41
DeBoer *et al*. [116]	2007	2, 4	healthy	10.50	NA	PC	FSIQ	WISC-III or WISC-IV	20	−0.55
DeBoer *et al*. [116]	2007	2, 4	patients	10.75	NA	PC	FSIQ	WISC-III or WISC-IV	21	0.25
DeBoer *et al*. [116]	2007	2, 4	patients	10.75	NA	PC	verbal	WISC-III or WISC-IV: VCI	21	0.30
DeBoer *et al*. [116]	2007	2, 4	healthy	10.50	NA	PC	verbal	WISC-III or WISC-IV: VCI	20	−0.20
DeBoer *et al*. [116]	2007	2, 4	patients	10.75	NA	PC	performance	WISC-III or WISC-IV: POI	21	0.38
DeBoer *et al*. [116]	2007	2, 4	healthy	10.50	NA	PC	performance	WISC-III or WISC-IV: POI	20	−0.22
Doernte [117]	2007	4	healthy	58.50	0.00	grey	FSIQ	HAWIE-R: sim, info, bd, pc	18	−0.23
Doernte [117]	2007	4	healthy	58.50	100.00	grey	FSIQ	HAWIE-R: sim, info, bd, pc	17	0.18
Doernte [117]	2007	4	patients	59.10	0.00	grey	FSIQ	HAWIE-R: sim, info, bd, pc	12	−0.02
Doernte [117]	2007	4	patients	59.10	100.00	grey	FSIQ	HAWIE-R: sim, info, bd, pc	23	−0.01
Fine *et al*. [118]	2007	2, 4	healthy	40.10	45.00	PC	FSIQ	WASI	44	−0.11
Fine *et al*. [118]	2007	2, 4	healthy	10.47	63.00	PC	FSIQ	WASI	24	0.23
Luders *et al*. [119]	2007	2, 3, 4	healthy	28.48	45.00	reported	FSIQ	WAIS-R	62	0.28
Nakamura *et al*. [120]	2007	2, 3, 4	healthy	40.80	90.00	PC	FSIQ	WAIS-III	44	0.38
Nakamura *et al*. [120]	2007	2, 4	patients	40.60	90.00	PC	FSIQ	WAIS-III	43	0.32
Nakamura *et al*. [120]	2007	2, 4	patients	40.60	90.00	PC	verbal	WAIS-III verbal	44	0.26
Nakamura *et al*. [120]	2007	2, 4	healthy	40.80	90.00	PC	verbal	WAIS-III verbal	44	0.40
Nakamura *et al*. [120]	2007	2, 4	patients	40.60	90.00	PC	performance	WAIS-III performance	44	0.34
Nakamura *et al*. [120]	2007	2, 4	healthy	40.80	90.00	PC	performance	WAIS-III performance	43	0.29
Narr *et al*. [121]	2007	4	healthy	28.24	46.20	reported	FSIQ	WAIS	63	0.36
Schottenbauer *et al*. [122]	2007	2, 3, 4	healthy	34.32	0.00	PC	FSIQ	WAIS-R	22	0.60
Schottenbauer *et al*. [122]	2007	2, 3, 4	healthy	37.77	100.00	PC	FSIQ	WAIS-R	35	0.33
Schottenbauer *et al*. [122]	2007	2, 4	patients	40.96	0.00	PC	FSIQ	WAIS-R	69	0.34
Schottenbauer *et al*. [122]	2007	2, 4	patients	39.64	100.00	PC	FSIQ	WAIS-R	205	0.28
Schottenbauer *et al*. [122]	2007	2, 4	patients	40.90	0.00	PC	verbal	WAIS-R: voc	68	0.43
Schottenbauer *et al*. [122]	2007	2, 4	healthy	34.32	0.00	PC	verbal	WAIS-R: voc	22	0.54
Schottenbauer *et al*. [122]	2007	2, 4	patients	39.66	100.00	PC	verbal	WAIS-R: voc	202	0.28
Schottenbauer *et al*. [122]	2007	2, 4	healthy	37.77	100.00	PC	verbal	WAIS-R: voc	35	0.38
Schottenbauer *et al*. [122]	2007	2, 4	patients	40.90	0.00	PC	performance	WAIS-R: bd	68	0.29
Schottenbauer *et al*. [122]	2007	2, 4	healthy	34.32	0.00	PC	performance	WAIS-R: bd	22	0.30
Schottenbauer *et al*. [122]	2007	2, 4	patients	39.65	100.00	PC	performance	WAIS-R: bd	203	0.17
Schottenbauer *et al*. [122]	2007	2, 4	healthy	37.77	100.00	PC	performance	WAIS-R: bd	35	0.17
Schumann *et al*. [123]	2007	2, 4	healthy	13.10	100.00	reported	FSIQ	WASI	22	0.41
Schumann *et al*. [123]	2007	2, 4	healthy	13.10	100.00	reported	verbal	WASI verbal	22	0.38
Schumann *et al*. [123]	2007	2, 4	healthy	13.10	100.00	reported	performance	WASI performance	22	0.25
Amat *et al*. [124]	2008	2, 3, 4	healthy	31.50	56.00	PC	FSIQ	WAIS-R	27	−0.11
Amat *et al*. [124]	2008	2, 4	healthy	31.50	56.00	PC	verbal	WAIS-R verbal	27	−0.29
Amat *et al*. [124]	2008	2, 4	healthy	31.50	56.00	PC	performance	WAIS-R performance	27	0.18
Choi *et al*. [125]	2008	4	healthy	21.60	54.30	reported	FSIQ	WAIS-R	164	0.35
Ebner *et al*. [126]	2008	2, 4	patients	34.52	68.00	PC	verbal	MWT-B	44	0.15
Ebner *et al*. [126]	2008	2, 4	healthy	32.45	51.00	PC	verbal	MWT-B	37	−0.13
Raz *et al*. [127]	2008	2, 4	healthy	51.11	43.00	PC	fluid	CFIT (form 2)	55	0.18
Raz *et al*. [127]	2008	2, 4	patients	59.75	25.00	PC	fluid	CFIT (form 2)	32	−0.02
Raz *et al*. [127]	2008	2, 4	patients	59.75	25.00	PC	verbal	Vocabulary Test (V2 & V3)	31	0.15
Raz *et al*. [127]	2008	2, 4	healthy	51.11	43.00	PC	verbal	Vocabulary Test (V2 & V3)	55	0.13
Castro-Fornieles *et al*. [128]	2009	2, 4	patients	14.50	8.00	PC	verbal	WISC-R: voc	12	0.11
Castro-Fornieles *et al*. [128]	2009	2, 4	healthy	14.60	11.00	PC	verbal	WISC-R: voc	9	0.43
Castro-Fornieles *et al*. [128]	2009	2, 4	patients	14.50	8.00	PC	performance	WISC-R: bd	12	0.38
Castro-Fornieles *et al*. [128]	2009	2, 4	healthy	14.60	11.00	PC	performance	WISC-R: bd	9	0.55
Miller *et al*. [129]	2009	2, 4	healthy	9.25	33.00	reported	FSIQ	WJIII (GIA)	12	0.23
Miller *et al*. [129]	2009	2, 4	healthy	12.08	NA	reported	fluid	WJIII: thinking ability	11	−0.11
Miller *et al*. [129]	2009	2, 4	patients	16.53	63.00	reported	FSIQ	WJIII (GIA)	16	−0.30
Miller *et al*. [129]	2009	2, 4	healthy	12.08	NA	reported	verbal	WJIII: Cog verbal ability	11	−0.65
Miller *et al*. [129]	2009	2, 4	healthy	9.25	NA	reported	verbal	WJIII: Cog verbal ability	5	0.84
Miller *et al*. [129]	2009	2, 4	patients	16.53	NA	reported	verbal	WJIII: Cog verbal ability	6	0.76
Qiu *et al*. [130]	2009	2, 4	healthy	10.50	53.00	PC	FSIQ	WISC-III or WISC-IV	66	0.26
Qiu *et al*. [130]	2009	2, 4	patients	10.40	57.00	PC	FSIQ	WISC-III or WISC-IV	47	0.26
Qiu *et al*. [130]	2009	2, 4	patients	10.40	57.00	PC	verbal	WISC-III or WISC-IV: VCI	47	0.21
Qiu *et al*. [130]	2009	2, 4	healthy	10.50	53.00	PC	verbal	WISC-III or WISC-IV: VCI	66	0.35
Qiu *et al*. [130]	2009	2, 4	patients	10.40	57.00	PC	performance	WISC-III or WISC-IV: POI	47	0.20
Qiu *et al*. [130]	2009	2, 4	healthy	10.50	53.00	PC	performance	WISC-III or WISC-IV: PRI	66	0.12
Shenkin *et al*. [131]	2009	2, 3, 4	healthy	78.40	29.00	reported	FSIQ	MHT, RSPM, Verbal fluency, lm	99	0.21
Shenkin *et al*. [131]	2009	2, 4	healthy	78.40	29.00	reported	verbal	COWA (verbal fluency)	107	0.13
Van Leeuwen *et al*. [132]	2009	2, 4	healthy	9.10	50.00	reported	fluid	RSPM	209	0.20
Van Leeuwen *et al*. [132]	2009	2, 4	healthy	9.10	50.00	reported	verbal	WISC-III: comp	209	0.33
Van Leeuwen *et al*. [132]	2009	2, 4	healthy	9.10	50.00	reported	performance	WISC-III: POI	209	0.28
Van Leeuwen *et al*. [132]	2009	4	healthy	9.10	50.00	reported	performance	WISC-III: PSI	209	0.12
Weniger *et al*. [133]	2009	2, 4	patients	32.00	0.00	PC	FSIQ	HAWIE-R	10	0.02
Weniger *et al*. [133]	2009	2, 3, 4	healthy	33.00	0.00	PC	FSIQ	HAWIE-R	25	0.15
Weniger *et al*. [133]	2009	2, 4	patients	32.00	0.00	PC	FSIQ	HAWIE-R	13	0.27
Weniger *et al*. [133]	2009	2, 4	patients	32.00	0.00	PC	verbal	HAWIE-R verbal	13	0.35
Weniger *et al*. [133]	2009	2, 4	healthy	33.00	0.00	PC	verbal	HAWIE-R verbal	25	0.00
Weniger *et al*. [133]	2009	2, 4	patients	32.00	0.00	PC	verbal	HAWIE-R verbal	10	−0.17
Weniger *et al*. [133]	2009	2, 4	patients	32.00	0.00	PC	performance	HAWIE-R performance	10	0.23
Weniger *et al*. [133]	2009	2, 4	healthy	33.00	0.00	PC	performance	HAWIE-R performance	25	0.24
Weniger *et al*. [133]	2009	2, 4	patients	32.00	0.00	PC	performance	HAWIE-R performance	13	0.16
Zeegers *et al*. [134]	2009	2, 4	patients	3.72	91.00	reported	FSIQ	unknown FS	21	0.06
Zeegers *et al*. [134]	2009	2, 4	patients	3.44	92.00	reported	FSIQ	unknown FS	10	0.73
Betjemann *et al*. [135]	2010	2, 4	healthy	11.40	52.00	reported	verbal	WISC-R verbal	142	0.14
Betjemann *et al*. [135]	2010	2, 4	healthy	11.40	52.00	reported	performance	WISC-R performance	142	0.42
Hermann [136]	2010	2, 3, 4	healthy	33.34	42.00	PC	FSIQ	Wechsler FS	67	0.31
Hermann [136]	2010	2, 4	patients	36.09	35.00	PC	FSIQ	Wechsler FS	77	0.21
Hermann [136]	2010	2, 4	patients	36.09	35.00	PC	verbal	Wechsler verbal	77	0.28
Hermann [136]	2010	2, 4	healthy	33.34	42.00	PC	verbal	Wechsler verbal	67	0.23
Hermann [136]	2010	2, 4	patients	36.09	35.00	PC	performance	Wechsler performance	77	0.09
Hermann [136]	2010	2, 4	healthy	33.34	42.00	PC	performance	Wechsler performance	67	0.33
Hogan *et al*. [137]	2010	2, 4	healthy	68.69	53.00	PC	fluid	RSPM	234	0.11
Hogan *et al*. [137]	2010	2, 4	healthy	68.69	53.00	PC	verbal	NART	235	0.00
Isaacs *et al*. [138]	2010	2, 4	healthy	15.75	0.00	PC	FSIQ	WISC-III or WAIS-III	24	0.00
Isaacs *et al*. [138]	2010	2, 4	healthy	15.75	100.00	reported	FSIQ	WISC-III or WAIS-III	26	0.36
Isaacs *et al*. [138]	2010	2, 4	healthy	15.75	0.00	PC	verbal	WISC-III or WAIS-III verbal	24	0.00
Isaacs *et al*. [138]	2010	2, 4	healthy	15.75	100.00	reported	verbal	WISC-III or WAIS-III verbal	26	0.48
Isaacs *et al*. [138]	2010	2, 4	healthy	15.75	0.00	PC	performance	WISC-III or WAIS-III performance	24	0.00
Isaacs *et al*. [138]	2010	2, 4	healthy	15.75	100.00	reported	performance	WISC-III or WAIS-III performance	26	0.19
Lange *et al*. [139]	2010	2, 4	healthy	10.88	0.00	reported	FSIQ	WASI	166	0.22
Lange *et al*. [139]	2010	2, 4	healthy	10.95	100.00	reported	FSIQ	WASI	143	0.23
Wallace *et al*. [140]	2010	2, 4	healthy	11.80	48.00	reported	FSIQ	WASI	649	0.14
Wallace *et al*. [140]	2010	2, 4	healthy	11.80	48.00	reported	verbal	WASI verbal	649	0.13
Wallace *et al*. [140]	2010	2, 4	healthy	11.80	48.00	reported	performance	WASI performance	649	0.14
Ashtari *et al*. [141]	2011	2, 3, 4	healthy	18.50	100.00	reported	FSIQ	WRAT-III	14	0.57
Ashtari *et al*. [141]	2011	2, 4	patients	19.30	100.00	reported	FSIQ	WRAT-III	14	0.29
Chen *et al*. [142]	2011	4	healthy	22.56	44.00	reported	FSIQ	WASI	27	0.02
Chen *et al*. [142]	2011	4	patients	23.07	46.70	reported	FSIQ	WASI	30	0.68
Chen *et al*. [142]	2011	4	patients	23.02	27.00	reported	FSIQ	WASI	37	0.41
Kievit *et al*. [143]	2011	2, 3, 4	healthy	21.10	36.00	PC	FSIQ	WAIS-III	80	0.29
Kievit *et al*. [143]	2011	2, 4	healthy	21.10	36.00	PC	verbal	WAIS-III verbal	80	0.23
Tate *et al*. [144]	2011	2, 4	patients	81.70	43.00	PC	FSIQ	Shipley	194	0.00
Aydin *et al*. [145]	2012	2, 4	healthy	15.10	100.00	reported	FSIQ	WISC-R	30	0.40
Aydin *et al*. [145]	2012	2, 4	healthy	15.10	100.00	reported	verbal	WISC-R verbal	30	0.26
Aydin *et al*. [145]	2012	2, 4	healthy	15.10	100.00	reported	performance	WISC-R performance	30	0.34
Burgaleta *et al*. [146]	2012	2, 3, 4	healthy	19.88	44.00	reported	FSIQ	9 tests from APM, DAT-AR-5, PMR-R	100	0.17
Bigler *et al*. [147]	2013	4	patients	10.66	58.00	reported	performance	WISC-IV: PSI	47	0.00
Bigler *et al*. [147]	2013	4	patients	10.67	68.00	reported	performance	WISC-IV: PSI	32	0.00
Bigler *et al*. [147]	2013	4	patients	10.14	58.00	reported	performance	WISC-IV: PSI	27	0.00
Royle *et al*. [148]	2013	2, 3, 4	healthy	72.47	100.00	reported	FSIQ	WAIS-III: ins, span, mr, bd, ss, ds	293	0.26
Royle *et al*. [148]	2013	2, 3, 4	healthy	72.60	0.00	reported	FSIQ	WAIS-III: ins, span, mr, bd, ss, ds	327	0.27
Royle *et al*. [148]	2013	4	healthy	72.47	100.00	reported	verbal	WAIS-III: lns	293	0.10
Royle *et al*. [148]	2013	4	healthy	72.60	0.00	reported	verbal	WAIS-III: lns	327	0.22
Royle *et al*. [148]	2013	4	healthy	72.47	100.00	reported	verbal	WAIS-III: span b	293	0.11
Royle *et al*. [148]	2013	4	healthy	72.60	0.00	reported	verbal	WAIS-III: span b	327	0.23
Royle *et al*. [148]	2013	4	healthy	72.47	100.00	reported	performance	WAIS-III: bd	293	0.25
Royle *et al*. [148]	2013	4	healthy	72.60	0.00	reported	performance	WAIS-III: bd	327	0.25
Royle *et al*. [148]	2013	4	healthy	72.47	100.00	reported	performance	WAIS-III: mr	293	0.14
Royle *et al*. [148]	2013	4	healthy	72.60	0.00	reported	performance	WAIS-III: mr	327	0.18
Royle *et al*. [148]	2013	4	healthy	72.47	100.00	reported	performance	WAIS-III: ds	293	0.22
Royle *et al*. [148]	2013	4	healthy	72.60	0.00	reported	performance	WAIS-III: ds	327	0.33
Royle *et al*. [148]	2013	4	healthy	72.47	100.00	reported	performance	WAIS-III: ss	293	0.17
Royle *et al*. [148]	2013	4	healthy	72.60	0.00	reported	performance	WAIS-III: ss	327	0.34
Zelko *et al*. [149]	2013	4	healthy	14.90	53.00	reported	FSIQ	WAIS or WISC: voc, sim, pc, bd, arith, ds, ss	36	0.25
Zelko *et al*. [149]	2013	4	patients	14.60	49.00	reported	FSIQ	WAIS or WISC: voc, sim, pc, bd, arith, ds, ss	108	0.23
Zelko *et al*. [149]	2013	4	patients	14.60	49.00	reported	verbal	WAIS or WISC: voc, sim	108	0.23
Zelko *et al*. [149]	2013	4	healthy	14.90	53.00	reported	verbal	WAIS or WISC: voc, sim	36	0.04
Zelko *et al*. [149]	2013	4	patients	14.60	49.00	reported	verbal	WAIS or WISC: arith, span	108	0.26
Zelko *et al*. [149]	2013	4	healthy	14.90	53.00	reported	verbal	WAIS or WISC: arith, span	36	0.33
Zelko *et al*. [149]	2013	4	patients	14.60	49.00	reported	performance	WAIS or WISC: pc, bd	108	0.21
Zelko *et al*. [149]	2013	4	healthy	14.90	53.00	reported	performance	WAIS or WISC: pc, bd	36	0.30
Zelko *et al*. [149]	2013	4	patients	14.60	49.00	reported	performance	WAIS or WISC: cod, sym	108	0.09
Zelko *et al*. [149]	2013	4	healthy	14.90	53.00	reported	performance	WAIS or WISC: cod, sym	36	−0.12
Bjuland *et al*. [150]	2014	4	healthy	20.30	42.00	reported	FSIQ	WAIS-III	60	0.36
Bjuland *et al*. [150]	2014	4	patients	20.10	41.00	reported	FSIQ	WAIS-III	43	0.56
Bjuland *et al*. [150]	2014	4	patients	20.30	41.00	reported	verbal	WAIS-III: VCI	42	0.44
Bjuland *et al*. [150]	2014	4	patients	20.30	41.00	reported	verbal	WAIS-III: WMI	42	0.54
Bjuland *et al*. [150]	2014	4	patients	20.30	41.00	reported	performance	WAIS-II: POI	43	0.48
Bjuland *et al*. [150]	2014	4	patients	20.30	41.00	reported	performance	WAIS-II: PSI	43	0.48
Grunewaldt *et al*. [151]	2014	4	patients	10.17	34.80	reported	FSIQ	WISC-III	21	0.00
Grunewaldt *et al*. [151]	2014	4	patients	10.17	34.80	reported	verbal	WISC-III: WMI	21	0.00
Jenkins *et al*. [152]	2014	4	healthy	11.70	41.70	reported	FSIQ	WASI, WISC-III or WPPSI-R: voc, mr	102	0.19
MacDonald *et al*. [153]	2014	4	healthy	11.60	100.00	reported	FSIQ	WASI	142	0.23
MacDonald *et al*. [153]	2014	4	healthy	11.30	0.00	reported	FSIQ	WASI	161	0.22
MacDonald *et al*. [153]	2014	4	healthy	11.60	100.00	reported	verbal	WASI verbal	142	0.13
MacDonald *et al*. [153]	2014	4	healthy	11.30	0.00	reported	verbal	WASI verbal	161	0.18
MacDonald *et al*. [153]	2014	4	healthy	11.60	100.00	reported	performance	WASI performance	142	0.29
MacDonald *et al*. [153]	2014	4	healthy	11.30	0.00	reported	performance	WASI performance	161	0.19
McCoy *et al*. [154]	2014	4	patients	13.00	100.00	reported	FSIQ	WISC-IV (GAI)	10	0.59
McCoy *et al*. [154]	2014	4	patients	13.00	0.00	reported	FSIQ	WISC-IV (GAI)	16	0.62
Zhu *et al*. [155]	2014	4	healthy	20.41	41.00	reported	FSIQ	WAIS-R (Chinese)	316	0.10
Boberg *et al*. [156]	2015	4	healthy	8.00	54.00	grey	FSIQ	WISC-IV (Swedish)	10	0.69
Boberg *et al*. [156]	2015	4	healthy	8.20	35.80	grey	FSIQ	WISC-IV (Swedish)	9	0.00
Boberg *et al*. [156]	2015	4	healthy	8.30	50.00	grey	FSIQ	WISC-IV	21	0.00
Grazioplene *et al*. [157]	2015	4	healthy	26.20	51.00	reported	FSIQ	WAIS-IV: voc, sim, mr, bd	285	0.28
Grazioplene *et al*. [157]	2015	4	healthy	23.50	100.00	reported	FSIQ	WAIS-IV: voc, sim, mr, bd	107	0.08
Grazioplene *et al*. [157]	2015	4	healthy	21.70	54.00	reported	FSIQ	WASI	125	0.04
Grazioplene *et al*. [157]	2015	4	healthy	26.20	51.00	reported	verbal	WAIS-IV: voc, sim	285	0.18
Grazioplene *et al*. [157]	2015	4	healthy	21.70	54.00	reported	verbal	WASI verbal	125	0.04
Grazioplene *et al*. [157]	2015	4	healthy	23.50	100.00	reported	verbal	WAIS-III: voc, sim	107	0.10
Grazioplene *et al*. [157]	2015	4	healthy	26.20	51.00	reported	performance	WAIS-IV: mr, bd	285	0.30
Grazioplene *et al*. [157]	2015	4	healthy	21.70	54.00	reported	performance	WASI performance	125	0.04
Grazioplene *et al*. [157]	2015	4	healthy	23.50	100.00	reported	performance	WAIS-III: mr, bd	107	0.04
Lefebvre *et al*. [158]	2015	4	healthy	17.00	83.00	reported	FSIQ	Wechsler	366	0.23
Lefebvre *et al*. [158]	2015	4	patients	16.60	88.00	reported	FSIQ	Wechsler	328	0.04
Lefebvre *et al*. [158]	2015	4	patients	16.60	88.00	reported	verbal	unknown verbal	241	0.08
Lefebvre *et al*. [158]	2015	4	healthy	17.00	83.00	reported	verbal	unknown verbal	297	0.22
Lefebvre *et al*. [158]	2015	4	patients	16.60	88.00	reported	performance	unknown performance	241	0.17
Lefebvre *et al*. [158]	2015	4	healthy	17.00	83.00	reported	performance	unknown performance	297	0.18
Paul *et al.* [159]	2015	4	healthy	24.57	0.00	reported	verbal	Reading Span, Rotation Span, Symmetrie Span	90	0.25
Paul *et al.* [159]	2015	4	healthy	24.07	100.00	reported	verbal	Reading Span, Rotation Span, Symmetrie Span	121	0.18
Walters *et al*. [160]	2015	4	patients	17.32	100.00	reported	FSIQ	WAIS or WISC: voc, mr	178	0.19
Ballester-Plane *et al*. [161]	2016	4	patients	25.10	67.00	reported	FSIQ	RCPM	30	0.73
Ballester-Plane *et al*. [161]	2016	4	patients	25.10	67.00	reported	verbal	PPVT-III	30	0.71
Ballester-Plane *et al*. [161]	2016	4	patients	25.10	67.00	reported	performance	WASI performance	30	0.72
Bohlken *et al*. [162]	2016	4	healthy	32.69	42.00	reported	FSIQ	WAIS-III: ds, bd, arith., span, inf	164	0.26
Bohlken *et al*. [162]	2016	4	healthy	32.70	42.00	reported	verbal	WAIS-III: inf	164	0.18
Bohlken *et al*. [162]	2016	4	healthy	32.70	42.00	reported	verbal	WAIS-III: arith	164	0.26
Bohlken *et al*. [162]	2016	4	healthy	32.70	42.00	reported	verbal	WAIS-III: span	164	0.00
Bohlken *et al*. [162]	2016	4	healthy	32.70	42.00	reported	performance	WAIS-III: bd	164	0.31
Bohlken *et al*. [162]	2016	4	healthy	32.70	42.00	reported	performance	WAIS-III: ds	164	0.12
Ferreira *et al*. [163]	2016	4	healthy	45.10	49.00	reported	verbal	WAIS-III: voc	73	0.36
Ferreira *et al*. [163]	2016	4	healthy	45.10	49.00	reported	verbal	WAIS-III: inf	73	0.50
Ferreira *et al*. [163]	2016	4	healthy	45.10	49.00	reported	performance	WAIS-III: bd	73	0.33
Gregory *et al*. [164]	2016	4	healthy	14.70	57.00	reported	FSIQ	Conditional Exclusion, Emotional Differentiation, Emotional Identification, Face Memory, Letter N-Back, Line Orientation, Matrix Reasoning, Verbal Reasoning, Visual Object Learning, Word Memory; WRAT: Reading	662	0.24
Monson *et al*. [165]	2016	4	patients	7.50	50.00	reported	FSIQ	WASI	134	0.26
Gregory *et al*. [164]	2016	4	patients	7.50	50.00	reported	verbal	WASI verbal	134	0.11
Gregory *et al*. [164]	2016	4	patients	7.50	50.00	reported	performance	WASI performance	134	0.31
Nikolaidis *et al*. [166]	2016	4	healthy	21.15	34.00	reported	fluid	RAPM, Shipley Abstraction, Letter Sets, Spatial Relations, Paper Folding, Form Boards	71	0.44
Nikolaidis *et al*. [166]	2016	4	healthy	21.15	34.00	reported	verbal	Visual Short-Term Memory, Spatial Working Memory, Running Span	71	0.13
Paul *et al*. [159]	2016	4	healthy	24.57	0.00	reported	fluid	BOMAT, Number Series, Letter Sets	90	0.14
Paul *et al*. [159]	2016	4	healthy	24.07	100.00	reported	fluid	BOMAT, Number Series, Letter Sets	121	0.13
Treit *et al*. [167]	2016	4	patients	12.50	53.00	reported	FSIQ	WRIT or WISC	50	0.21
Treit *et al*. [167]	2016	4	healthy	11.90	48.00	reported	FSIQ	WRIT or WISC	66	0.09
Amaral *et al*. [168]	2017	4	healthy	3.00	100.00	reported	FSIQ	MSEL	49	0.35
Amaral *et al*. [168]	2017	4	patients	3.08	100.00	reported	FSIQ	MSEL	19	−0.18
Amaral *et al*. [168]	2017	4	patients	3.13	100.00	reported	FSIQ	MSEL	110	0.01
Arhan *et al*. [169]	2017	4	healthy	9.20	46.00	reported	FSIQ	WISC-R (Turkish)	46	0.51
Arhan *et al*. [169]	2017	4	healthy	9.20	46.00	reported	verbal	WISC-R: voc	46	0.71
Arhan *et al*. [169]	2017	4	healthy	9.20	46.00	reported	verbal	WISC-R: sim	46	0.54
Arhan *et al*. [169]	2017	4	healthy	9.20	46.00	reported	verbal	WISC-R: inf	46	0.38
Arhan *et al*. [169]	2017	4	healthy	9.20	46.00	reported	verbal	WISC-R: comp	46	0.45
Arhan *et al*. [169]	2017	4	healthy	9.20	46.00	reported	verbal	WISC-R: arith	46	0.24
Arhan *et al*. [169]	2017	4	healthy	9.20	46.00	reported	verbal	WISC-R: span	46	0.31
Arhan *et al*. [169]	2017	4	healthy	9.20	46.00	reported	performance	WISC-R: pc	46	0.77
Arhan *et al*. [169]	2017	4	healthy	9.20	46.00	reported	performance	WISC-R: pic	46	0.24
Arhan *et al*. [169]	2017	4	healthy	9.20	46.00	reported	performance	WISC-R: bd	46	0.04
Arhan *et al*. [169]	2017	4	healthy	9.20	46.00	reported	performance	WISC-R: obj	46	0.04
Arhan *et al*. [169]	2017	4	healthy	9.20	46.00	reported	performance	WISC-R: ds	46	0.27
Martinez *et al*. [170]	2017	4	healthy	19.60	0.00	reported	verbal	DAT-VR, PMA-V, Reading Span, Letter Memory, Keep Track, Flanker (verbal + numerical), Simple Recognition verbal task	40	0.28
Martinez *et al*. [170]	2017	4	healthy	20.20	100.00	reported	verbal	DAT-VR, PMA-V, Reading Span, Letter Memory, Keep Track, Flanker (verbal + numerical), Simple Recognition verbal task	40	−0.04
Martinez *et al*. [170]	2017	4	healthy	19.60	0.00	reported	spatial	DAT-SR, PMA-S, Rotation of Solid Figures, Dot Matrix, 2-Backl task, spatial Simon task, spatial Simple Recognition	40	0.39
Martinez *et al*. [170]	2017	4	healthy	20.20	100.00	reported	spatial	DAT-SR, PMA-S, Rotation of Solid Figures, Dot Matrix, 2-Backl task, spatial Simon task, spatial Simple Recognition	40	0.24
Ritchie *et al*. [171]	2017	4	healthy	92.10	45.00	reported	fluid	WAIS-III: ds, WMS-R: Logical Memory Story A, Phonemic Verbal Fluency [172]	34	0.23
Ritchie *et al*. [171]	2017	4	healthy	92.10	45.00	reported	performance	WAIS-III: ds	34	0.19
van der Linden *et al*. [173]	2017	4	healthy	28.82	0.00	reported	FSIQ	Penn Progressive Matrices, Peabody Vocabulary, Oral Reading Recognition, List Sorting, Picture Sequence Memory, Penn Line Orientation, Dimensional Change Card Sorting, Word Memory, Salthouse Pattern Comparison, Flanker Task	503	0.26
van der Linden *et al*. [173]	2017	4	healthy	28.82	100.00	reported	FSIQ	Penn Progressive Matrices, Peabody Vocabulary, Oral Reading Recognition, List Sorting, Picture Sequence Memory, Penn Line Orientation, Dimensional Change Card Sorting, Word Memory, Salthouse Pattern Comparison, Flanker Task	393	0.25
Vreeker *et al*. [174]	2017	4	healthy	44.60	49.00	reported	FSIQ	WAIS-III (Dutch): info, srith., bf, ds	160	0.28
Annink *et al*. [175]	2018	4	patients	9.79	48.00	reported	FSIQ	WISC-III (Dutch)	52	0.43
Jensen *et al*. [176]	2018	4	healthy	24.91	59.00	PC	FSIQ	WAIS-III (Danish): voc, sim, bd, mr	56	0.30
Jensen *et al*. [176]	2018	4	patients	24.69	57.40	PC	FSIQ	WAIS-III (Danish): voc, sim, bd, mr	54	0.14
Jensen *et al*. [176]	2018	4	healthy	24.91	59.00	PC	verbal	WAIS-III (Danish): voc, sim	56	0.30
Jensen *et al*. [176]	2018	4	patients	24.69	57.40	PC	verbal	WAIS-III (Danish): voc, sim	54	0.09
Jensen *et al*. [176]	2018	4	healthy	24.91	59.00	PC	performance	WAIS-III (Danish): bd, mr	56	0.19
Jensen *et al*. [176]	2018	4	patients	24.69	57.40	PC	performance	WAIS-III (Danish): bd, mr	54	0.20
Lammers *et al*. [177]	2018	4	patients	72.00	61.00	reported	FSIQ	Paired Associate Learning, Verbal Recognition Memory, Spatial Span Length, Simple Reaction Time, TrailAB, Grooved Pegboard task	243	0.18
Mankovsky *et al*. [178]	2018	4	patients	62.30	33.00	reported	verbal	RAVLT: immediate recall, delayed recall, delayed recognition + WAIS-III: span	93	0.02
Mankovsky *et al*. [178]	2018	4	patients	62.30	33.00	reported	performance	Trails (A), SCWT (I + II), WAIS-II: ds	93	0.08
Nygaard *et al*. [179]	2018	4	patients	18.96	60.00	reported	FSIQ	WASI: voc, mr	82	0.30
Sreedharan *et al*. [180]	2018	4	patients	10.80	66.00	reported	FSIQ	WISC (Malayalam translation)	30	0.00
Takeuchi *et al*. [181]	2018	4	healthy	20.80	58.00	reported	FSIQ	Tanaka B Intelligence Scale	1319	0.07
Tozer *et al*. [182]	2018	4	patients	70.01	65.00	reported	FSIQ	span, lm, Visual Reproduction, BIRT Memory and Information Processing Battery, Speed of Information Processing, ds, Grooved Pegboard, trails, Verbal Fluency, WCST	118	0.23
Tozer *et al*. [182]	2018	4	patients	70.01	65.00	reported	performance	unknown	115	0.28
Ahn *et al*. [183]	2019	4	patients	32.97	42.00	reported	FSIQ	K-WAIS-R	38	0.00
Ahn *et al*. [183]	2019	4	patients	32.97	42.00	reported	verbal	WAIS-R verbal	38	0.00
Ahn *et al*. [183]	2019	4	patients	32.97	42.00	reported	performance	WAIS-R performance	38	0.00
Bathelt *et al*. [184]	2019	4	healthy	9.93	54.00	reported	FSIQ	WASI-II: Reasoning; AWMA: Digit Recall, Backward Digit Recall, Dot Matrix, Mr X	63	0.07
Bathelt *et al*. [184]	2019	4	patients	9.35	64.70	reported	FSIQ	WASI-II: Reasoning; AWMA: Digit Recall, Backward Digit Recall, Dot Matrix, Mr X	139	0.02
Cox *et al*. [185]	2019	4	healthy	63.13	100.00	reported	FSIQ	composite of 4 tests	3900	0.21
Cox *et al*. [185]	2019	4	healthy	63.13	0.00	reported	FSIQ	composite of 4 tests	4192	0.26
de Zwarte *et al.* [186]	2019	4	patients	27.49	60.00	reported	FSIQ	WAIS-III (Dutch): inf, arith, bd, ds	516	0.29
de Zwarte *et al.* [186]	2019	4	patients	52.85	32.00	reported	FSIQ	GIT (short)	85	0.06
Elliott *et al*. [187]	2019	4	healthy	45.00	48.00	reported	FSIQ	WAIS-IV	596	0.35
Elliott *et al*. [187]	2019	4	healthy	22.23	47.00	reported	FSIQ	Shipley	1163	0.12
Elliott *et al*. [187]	2019	4	healthy	20.26	47.00	reported	FSIQ	WASI: voc, mr	515	0.16
Hiraiwa *et al*. [188]	2019	4	patients	9.43	52.00	reported	FSIQ	WISC-IV	27	0.34
van Haren *et al*. [189]	2019	4	healthy	12.74	53.00	reported	FSIQ	WISC-III or WAIS-III: voc, inf, bd, pic	40	0.34
van Haren *et al*. [189]	2019	4	patients	13.77	30.00	reported	FSIQ	WISC-III or WAIS-III: voc, inf, bd, pic	40	0.53
van Haren *et al*. [189]	2019	4	patients	14.52	56.00	reported	FSIQ	WISC-III or WAIS-III: voc, inf, bd, pic	63	0.39
Cadenas-Sanchez *et al.* [190]	2020	4	patients	10.00	60.00	reported	FSIQ	K-BIT	100	−0.03
Cadenas-Sanchez *et al.* [190]	2020	4	patients	10.00	60.00	reported	verbal	Delayed non-Match to Sample Task	100	0.06
Corley *et al.* [191]	2020	4	healthy	79.40	53.10	reported	FSIQ	WAIS-III: mr, bd, ss, ds; WMS-III: spatial span f + b, vpa, lm; Four-Choice Reaction Time, Visual Inspection Time, NART, WTAR, PVF	358	0.19
Corley *et al.* [191]	2020	4	healthy	79.40	53.10	reported	verbal	NART, WTAR, PVF	358	0.08
Corley *et al.* [191]	2020	4	healthy	79.40	53.10	reported	verbal	WMS-III: vpa, lm; WAIS-III: ds (b)	358	0.16
Corley *et al.* [191]	2020	4	healthy	79.40	53.10	reported	performance	WAIS-III: mr, bd; WMS-III: spatial span f + b	358	0.12
Corley *et al.* [191]	2020	4	healthy	79.40	53.10	reported	performance	WAIS-III: ss, ds; Four Choice Reaction Time, Visual Inspection Time	358	0.29
de Zwarte *et al.* [186]	2020	4	patients	32.14	46.00	reported	FSIQ	mostly abbreviated Wechsler Scales	968	0.20
de Zwarte *et al.* [186]	2020	4	patients	27.48	41.00	reported	FSIQ	mostly abbreviated Wechsler Scales	507	0.20
Elias [192]	2020	4	patients	69.02	100.00	grey	verbal	WTAR	49	0.35
Laliberté Durish [193]	2020	4	patients	12.30	62.40	grey	FSIQ	WASI-II	304	0.19
Laliberté Durish [193]	2020	4	patients	12.50	51.90	grey	FSIQ	WASI-II	161	−0.07
Laliberté Durish [193]	2020	4	healthy	39.60	43.00	reported	FSIQ	Verbal Learning, ds, Conditional Exclusion, Spatial Working Memory, Facial Memory, TrailAB, Continuous Performance, Letter-Number Seq, Balloon Analog, Oral Word Association, Emotional Recognition	1216	0.12
Mitchell *et al*. [194]	2020	4	healthy	22.30	38.00	reported	FSIQ	MAB (5 subtests); WAIS-R: ds	1097	0.25
Williams *et al.* [195]	2020	4	patients	14.54	87.74	reported	FSIQ	Wechsler	302	0.08
Williams *et al.* [195]	2020	4	healthy	14.54	80.40	reported	FSIQ	Wechsler	352	0.31
Yankowitz *et al.* [196]	2020	4	healthy	13.10	72.70	reported	FSIQ	DAS (GCA) or WISC-IV or WASI (I or II)	216	0.38
Yankowitz *et al.* [196]	2020	4	patients	13.00	82.10	reported	FSIQ	DAS (GCA) or WISC-IV or WASI (I or II)	240	0.05
Yankowitz *et al.* [196]	2020	4	healthy	20.60	100.00	reported	FSIQ	WISC-III or WASI-I or WAIS (R or III)	89	0.35
Yankowitz *et al.* [196]	2020	4	patients	16.60	100.00	reported	FSIQ	WISC-III or WASI-I or WAIS (R or III)	86	0.25
Hedderich *et al.* [197]	2021	4	patients	26.70	57.40	reported	FSIQ	abb. WAIS-III (German)	97	0.38
Naef *et al.* [198]	2021	4	patients	26.71	38.60	reported	FSIQ	short form of WAIS-IV	44	0.14

## 3. Results

A summary effect of *r* = 0.23 for full-scale IQ was observed (*k* = 194; *I*^2^ = 60.70; 95% *CI* [0.21; 0.26]), when all available independent effect sizes were synthesized by means of the Hedges & Olkin approach. Summary effects were somewhat smaller when associations were limited to verbal (*r* = 0.20; *k* = 115; *I*^2^ = 43.84; 95% *CI* [0.16; 0.23]) or performance IQ domains (*r* = 0.20; *k* = 82; *I*^2^ = 27.66; 95% *CI* [0.17; 0.24]).

The Hunter & Schmidt-typed synthesis of artefact-corrected coefficients was broadly consistent with the results of the Hedges & Olkin approach, but unsurprisingly yielded somewhat larger effects for full-scale (*r* = 0.26; *k* = 116; *I*^2^ = 61.28; 95% *CI* [0.21; 0.031]), verbal (*r* = 0.22; *k* = 50; *I*^2^ = 50.28; 95% *CI* [0.15; 0.29]) and performance IQ (*r* = 0.25; *k* = 45; *I*^2^ = 30.42; 95% *CI* [0.20; 0.31]). Of note, these analyses were based on fewer observations compared to the other approaches, because necessary information for corrections (e.g. within-sample standard deviations of IQ scores) had not been reported. Results from the robust variance estimation-based approach were virtually identical with the Hedges & Olkin analyses showing small-to-moderate associations for full-scale (*r* = 0.23; *k* = 203; *I*^2^ = 54.85; 95% *CI* [0.20; 0.25]), verbal (*r* = 0.21; *k* = 141; *I*^2^ = 48.34; 95% *CI* [0.18; 0.24]) and performance IQ (*r* = 0.21; *k* = 110; *I*^2^ = 32.41; 95% *CI* [0.18; 0.24]).

**Figure 2.**
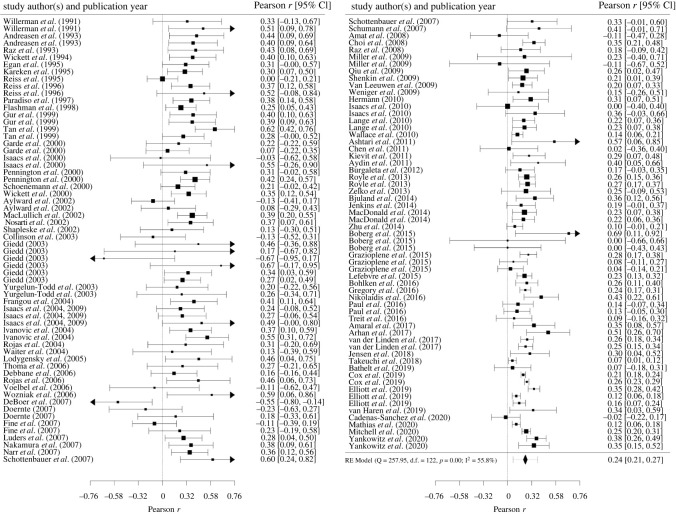
Forest plot of healthy samples (Hedges & Olkin model).

**Figure 3.**
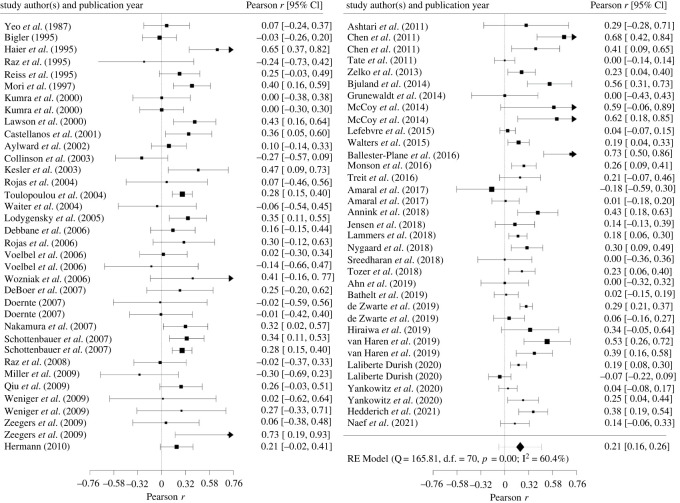
Forest plot of patient samples (Hedges & Olkin model).

This pattern of results remained virtually identical when analyses were limited to healthy (neurotypical) samples, although effect sizes were somewhat larger in this subgroup ranging from *r* = 0.19 to 0.24 for the Hedges & Olkin, 0.23 to 0.29 for the Hunter & Schmidt, and 0.20 to 0.24 for the RVE method (left side of table 2; figure 2 for a forest plot of the Hedges & Olkin analysis). Patient samples also showed non-trivial small-to-moderate effects, although they were slightly weaker than those of healthy samples (excepting Hedges & Olkin, as well as RVE analyses for verbal IQ; right part of table 2; figure 3 for a forest plot of the Hedges & Olkin-typed analysis).

**Table 2.** Summary effects of healthy and patient samples according to three different analysis approaches. Note. In the RVE approach, the number of synthesized effect sizes is followed by the number of independent samples in parentheses; *I*^2^ = percentage of variability due to variability of true effects; LCI = lower bound of 95% confidence interval; UCI = upper bound of 95% confidence interval.

	healthy samples						patient samples					
	*k*	*n*	*I*^2^	*r*	LCI	UCI	*k*	*n*	*I*^2^	*r*	LCI	UCI
full-scale IQ												
Hedges & Olkin approach	123	23 403	55.75	0.24	0.22	0.27	71	5361	60.43	0.21	0.16	0.26
psychometric meta-analysis	69	9057	32.76	0.29	0.24	0.33	47	3773	72.11	0.20	0.11	0.29
robust variance estimation (RVE)	128 (121)	24 543	53.04	0.24	0.22	0.27	75 (69)	7185	56.34	0.20	0.15	0.27
verbal IQ												
Hedges & Olkin approach	70	5440	47.47	0.19	0.14	0.23	45	2237	35.95	0.22	0.16	0.28
psychometric meta-analysis	31	2349	44.66	0.23	0.15	0.31	19	948	52.33	0.19	0.06	0.32
robust variance estimation (RVE)	93 (75)	8262	52.65	0.20	0.16	0.25	48 (46)	2550	39.32	0.22	0.16	0.28
performance IQ												
Hedges & Olkin approach	49	4162	31.96	0.21	0.17	0.25	33	1858	23.66	0.19	0.12	0.25
psychometric meta-analysis	28	2192	15.23	0.28	0.22	0.33	17	879	35.30	0.20	0.08	0.32
robust variance estimation (RVE)	74 (59)	8095	31.69	0.22	0.19	0.26	36 (32)	2078	33.90	0.19	0.13	0.25

Results of leave-one-out analyses did not show any meaningful influences of single leverage points on effect-size estimations for all three IQ domains in overall analyses or subsets. Outlier analyses revealed a maximum of three potential leverage points in the datasets (see, electronic supplementary material, S1). Because recalculations of effect estimates when omitting these data points did not lead to meaningful changes in summary effect calculations (the largest observed influence of any of these outliers led to changes in the third decimal place of the summary effect in either domain), all subsequent analyses were performed without excluding these data points.

### 3.1. Subgroup analyses

No significant group differences were observed between healthy and patient-based samples in full-scale (*k* = 194; *Q*(1) = 1.06; *p* = 0.304), verbal (*k* = 115; *Q*(1) = 0.75; *p* = 0.385) or performance IQ effects (*k* = 82; *Q*(1) = 0.41; *p* = 0.520). However, we assessed healthy (neurotypical) and patient-based samples separately in the subsequent subgroup analyses to allow meaningful comparisons with the results of previous accounts (i.e. [23–25]).

Positive meaningful associations between IQ and brain volume were observable within all investigated subsets (i.e. regardless of sample type: healthy versus patient samples or IQ domain: full-scale versus verbal versus performance IQ; excepting a trivial positive full-scale IQ and brain volume associations for grey literature findings in patient; note that another three out of a total of 65 summary effects failed to reach nominal significance), thus generalizing across publication status, sample age, volumetric measurement type (i.e. total brain versus intracranial volume), sex, and *g-*ness (see, table 3). Somewhat numerically larger effects were consistently observed in healthy compared to patient samples for full-scale and performance, but not for verbal IQ.

**Table 3.** Subgroup analyses for full-scale, verbal, and performance IQ. Note. Grey literature results were excluded from subgroup calculations for verbal and performance IQ because of low cell frequencies; TBV = Total brain volume; ICV = intracranial volume.

	healthy samples						patient samples					
	*k*	*n*	*I*^2^	*r*	LCI	UCI	*k*	*n*	*I*^2^	*r*	LCI	UCI
full-scale IQ												
total	123	23 403	55.75	0.24	0.22	0.27	71	5361	60.43	0.21	0.16	0.26
publication status	*k* = 123; *Q*(2) = 2.08; *p* = 0.353						*k* = 71; *Q*(2) = 6.72; *p* = 0.035					
reported	74	21 826	66.78	0.25	0.22	0.28	47	3842	67.97	0.24	0.18	0.31
grey literature	5	75	24.26	0.10	−0.34	0.50	4	500	58.89	0.05	−0.17	0.27
personal communication	44	1502	22.54	0.21	0.14	0.28	20	1019	30.04	0.17	0.09	0.25
age	*k* = 123; *Q*(1) < 0.01; *p* = 0.969						*k* = 71; *Q*(1) = 2.50; *p* = 0.114					
children/adolescents	56	4326	33.30	0.24	0.19	0.29	36	2569	54.46	0.17	0.10	0.24
adults	67	19 077	65.72	0.24	0.21	0.28	35	2792	61.47	0.25	0.18	0.32
volumetric measurement type	*k* = 94; *Q*(1) = 0.91; *p* = 0.339						*k* = 62; *Q*(1) = 3.34; *p* = 0.068					
TBV	78	17 891	48.86	0.24	0.21	0.27	45	3913	58.16	0.19	0.13	0.25
ICV	16	4448	80.08	0.28	0.19	0.36	17	1077	68.40	0.31	0.18	0.43
sex	*k* = 60; *Q*(1) = 0.10; *p* = 0.751						*k* = 18; *Q*(1) = 4.44; *p* = 0.035					
men	36	6137	12.91	0.26	0.22	0.29	12	735	22.15	0.16	0.06	0.26
women	24	5994	0.02	0.26	0.23	0.29	6	160	<0.01	0.33	0.15	0.49
*g-*ness	*k* = 106; *Q*(2) = 23.69; *p* < 0.001						*k* = 63; *Q*(2) = 0.38; *p* = 0.829					
fair *g-*ness	7	563	59.00	0.33	0.16	0.47	2	62	91.94	0.42	−1.00	1.00
good *g-*ness	48	18 309	56.70	0.19	0.16	0.22	30	3045	62.32	0.20	0.13	0.27
excellent *g-*ness	51	3364	4.07	0.31	0.27	0.34	31	1404	45.86	0.22	0.14	0.30
verbal IQ												
total	70	5440	47.47	0.19	0.14	0.23	45	2237	35.95	0.22	0.15	0.28
publication status	*k* = 70; *Q*(1) = 0.85; *p* = 0.358						*k* = 44; *Q*(1) = 2.39; *p* = 0.302					
reported	42	4336	54.48	0.20	0.14	0.25	21	1316	62.36	0.26	0.15	0.37
grey literature	—	—	—	—	—	—	1	49	—	0.35	0.08	0.58
personal communication	28	1104	33.79	0.15	0.07	.24	23	872	14.68	0.19	0.11	0.26
age	*k* = 70; *Q*(1) = 0.49; *p* = 0.484						*k* = 45; *Q*(1) = 7.11; *p* = 0.008					
children/adolescents	26	2131	28.06	0.21	0.14	0.28	13	693	0.01	0.11	0.02	0.20
adults	44	3309	51.64	0.18	0.12	0.23	32	1544	33.84	0.25	0.18	0.32
volumetric measurement type	*k* = 58; *Q*(1) = 3.47; *p* = 0.062						*k* = 36; *Q*(1) = 4.19; *p* = 0.004					
TBV	45	4296	40.38	0.17	0.12	0.21	23	1213	27.72	0.17	0.08	0.25
ICV	13	765	64.64	0.30	0.15	0.43	13	756	54.60	0.31	0.19	0.43
sex	*k* = 35; *Q*(1) = 0.08; *p* = 0.772						*k* = 15; *Q*(1) = 2.36; *p* = 0.125					
men	21	1021	28.08	0.23	0.15	0.31	5	126	<0.01	0.38	0.12	0.60
women	14	632	<0.01	0.25	0.17	0.32	10	443	<0.01	0.23	0.13	0.32
Performance IQ												
total	49	4162	31.96	0.21	0.17	0.25	33	1858	23.66	0.19	0.12	0.25
publication status	*k* = 49; *Q*(1) = 2.65; *p* = 0.104						*k* = 33; *Q*(1) = 0.81; *p* = 0.370					
reported	28	3549	50.32	0.23	0.18	0.28	16	1135	54.52	0.21	0.10	0.32
grey literature	—	—	—	—	—	—	—	—	—	—	—	—
personal communication	21	613	<0.01	0.17	0.10	0.23	17	1206	<0.01	0.16	0.08	0.23
age	*k* = 49; *Q*(1) = 0.53; *p* = 0.469						*k* = 33; *Q*(1) = 1.84; *p* = 0.175					
children/adolescents	23	2075	32.43	0.23	0.16	0.29	13	767	24.61	0.14	0.03	0.24
adults	26	2087	29.68	0.20	0.15	0.25	20	1091	36.90	0.22	0.13	0.31
volumetric measurement type	*k* = 38; *Q*(1) = 2.33; *p* = 0.127						*k* = 25; *Q*(1) = 0.708; *p* = 0.400					
TBV	32	3700	43.73	0.21	0.16	0.26	16	1012	<0.01	0.16	0.09	0.23
ICV	6	180	<0.01	0.29	0.17	0.40	9	609	79.25	0.26	<0.01	0.48
sex	*k* = 25; *Q*(1) = 0.05; *p* = 0.820						*k* = 9; *Q*(1) = 5.01; *p* = 0.025					
men	16	717	17.44	0.24	0.16	0.31	6	330	6.21	0.11	−0.07	0.28
women	9	429	<0.01	0.25	0.17	0.32	3	91	<0.01	0.27	0.13	0.40

There was only one significant difference between subset summary effects in healthy participants, indicating stronger associations with tests that were deemed to possess excellent compared to those that possess good *g-*ness. For patient samples, publication type in full-scale IQ, volumetric measurement in verbal IQ, and sex in performance IQ showed significantly larger effects for published, intracranial volume and female samples. However, these patient-based findings should be taken with a grain of salt because of low sample numbers (for details, see rightmost columns of table 3). Of note, between-studies variation remained moderate-to-large according to widely accepted classifications (0–25% suggest trivial, 25–50% small, 50–75% moderate and 75–100% large heterogeneity; see [199]), indicating potential further sources for explaining unobserved heterogeneity.

### 3.2. Meta-regressions

#### 3.2.1. Single regressions

Primary study publication years negatively predicted effect sizes of brain size associations with full-scale IQ in the total and healthy samples (*b*s = −0.004 and −0.005; *p*s = 0.028 and 0.006, respectively), but not in patient samples (*b* < 0.001; *p* = 0.979). In the verbal and performance IQ domains, publication years again showed consistently negative influences on associations in total and healthy samples, although associations failed to reach nominal statistical significance (*b*s range: −0.006 to ≥0.001; *p* range: 0.055 to 0.883; effect declines for healthy samples by domain are illustrated in the electronic supplementary material, S1). Regression coefficients for patient samples were inconsistent in signs (*p*s > 0.475).

In our RVE-based comparisons of intelligence domains for our total samples (i.e. full-scale versus verbal versus performance IQ), no significant differences were observable between associations (all *p*s > 0.05, when referencing to full-scale IQ). When removing the intercept from the model, regression coefficients yielded similar values for full-scale, performance and verbal IQ (*r*s = 0.22, 0.21 and 0.23, respectively; all *p*s < 0.001). These outcomes were similar when running regressions on either healthy or patient samples only. No significant domain differences in association strength were observed in either group (all *p*s > 0.05) and once more full-scale, verbal and performance IQ showed consistently significant coefficients for healthy and patient samples (*r*s = 0.24 and 0.20, 0.20 and 0.23, 0.24 and 0.18, respectively).

#### 3.2.2. Multiple regressions

In our hierarchical multiple meta-regressions, *g-*ness, primary study publication years and primary study publication status emerged as the most meaningful predictors of associations with full-scale IQ in healthy samples in our first block, explaining 46.18% of variance. Positive associations with *g-*ness indicated stronger effects for more highly *g-*loaded tests, thus corroborating our results from the subgroup analyses. Negative associations with publication years pointed towards declining effect sizes over time and effects were bigger when they had been published than when they had been obtained from the grey literature or personal communications. Neither addition of male ratio and mean age in our second nor study goal and number of corrections within studies in our third block led to improved model fit (top left of table 4).

**Table 4.** Theory-guided multiple hierarchical meta-regressions by sample type and IQ domain. Note. *SE b* = standard error of regression coefficient; *g-*ness: 0 = fair/good, 1 = excellent; NoC = Number of controlled variables; Publication status: 0 = published, 1 = unpublished (i.e. obtained from grey literature or personal communications); Study goal: 0 = report of correlation was not focus of study, 1 = report of correlation was focus of study; if likelihood ratio tests of block 2 versus block 1 did not yield significant results, block 3 was compared with block 1; all *VIF*s < 1.88.

	healthy samples					patient samples				
	*B*	*LCI*	*UCI*	*SE b*	*p*	*B*	*LCI*	*UCI*	*SE b*	*p*
full-scale IQ										
block 1	*k* = 104; *R*^2^ = 46.18					*k* = 62; *R*^2^ = 15.72				
* g-*ness	0.064	0.020	0.109	0.023	0.005	0.027	−0.104	0.158	0.065	0.680
publication year	−0.008	−0.012	−0.005	0.002	<0.001	0.001	−0.008	0.010	0.004	0.781
publication status	−0.096	−0.172	−0.020	0.038	0.014	−0.124	−0.250	0.003	0.063	0.055
block 2	*k* = 104; *R*^2^ = 58.94; *χ*^2^(2) = 2.179; *p* = 0.336					*k* = 62; *R*^2^ = 25.09; *χ*^2^(2)=4.013; *p* = 0.135				
* g-*ness	0.069	0.024	0.113	0.022	0.003	0.028	−0.103	0.159	0.065	0.666
publication year	−0.008	−0.012	−0.005	0.002	<0.001	0.001	−0.007	0.010	0.004	0.753
publication status	−0.098	−0.175	−0.021	0.039	0.013	−0.115	−0.240	0.010	0.062	0.070
male ratio	−0.016	−0.086	0.053	0.035	0.647	−0.212	−0.446	0.023	0.117	0.076
mean age	0.001	≥0.001	0.002	0.001	0.121	−0.001	−0.004	0.002	0.002	0.650
block 3	*k* = 104; *R*^2^ = 58.73; *χ*^2^(4)=4.791; *p* = 0.310					*k* = 62; *R*^2^ = 36.67; *χ*^2^(4)=9.894; *p* = 0.042				
* g-*ness	0.066	0.022	0.110	0.022	0.004	0.058	−0.079	0.196	0.069	0.399
publication year	−0.007	−0.011	−0.003	0.002	0.001	0.001	−0.008	0.010	0.004	0.797
publication status	−0.092	−0.178	−0.006	0.043	0.037	−0.107	−0.232	0.018	0.062	0.091
male ratio	−0.014	−0.083	0.056	0.035	0.694	−0.267	−0.502	−0.032	0.117	0.027
mean age	0.001	−0.001	0.002	0.001	0.299	≥0.001	−0.004	0.003	0.002	0.908
study goal	−0.019	−0.074	0.037	0.028	0.509	−0.117	−0.247	0.013	0.065	0.078
NoC	−0.014	−0.034	0.006	0.010	0.162	0.020	−0.031	0.071	0.025	0.443
verbal IQ										
block 1	*k* = 67; *R*^2^ = 10.30					*k* = 41; *R*^2^ = <0.01				
publication year	−0.006	−0.012	≥0.001	0.003	0.046	−0.002	−0.010	0.007	0.004	0.639
publication status	−0.049	−0.144	0.047	0.048	0.313	−0.038	−0.166	0.090	0.063	0.552
block 2	*k* = 67; *R*^2^ = 43.72; *χ*^2^(2) = 5.024; *p* = 0.081					*k* = 41; *R*^2^ = 26.81; *χ*^2^(2) = 2.173; *p* = 0.337				
publication year	−0.006	−0.011	≥0.001	0.003	0.039	−0.002	−0.010	0.007	0.004	0.658
publication status	−0.036	−0.129	0.057	0.047	0.443	−0.031	−0.162	0.100	0.065	0.629
male ratio	−0.008	−0.131	0.116	0.062	0.901	−0.070	−0.293	0.153	0.110	0.527
mean age	−0.002	−0.004	≥0.001	0.001	0.025	0.002	−0.002	0.006	0.002	0.291
block 3	*k* = 67; *R*^2^ = 74.19; χ²(4)=13.649; *p* = 0.009					*k* = 41; *R*^2^ = 39.13; χ²(4)=4.083; *p* = 0. 395				
publication year	−0.006	−0.011	−0.001	0.003	0.022	−0.004	−0.014	0.006	0.005	0.466
publication status	−0.086	−0.185	0.014	0.050	0.091	0.029	−0.133	0.191	0.080	0.718
male ratio	−0.027	−0.145	0.091	0.059	0.651	−0.061	−0.287	0.166	0.111	0.590
mean age	−0.001	−0.003	<0.001	0.001	0.120	0.002	−0.002	0.006	0.002	0.315
study goal	0.045	−0.042	0.132	0.044	0.308	−0.066	−0.246	0.114	0.089	0.463
noc	−0.051	−0.087	−0.015	0.018	0.006	0.032	−0.028	0.092	0.030	0.290
performance IQ										
block 1	*k* = 47; *R*^2^ < 0.01					*k* = 31; *R*2 = 41.92				
publication year	−0.003	−0.008	0.003	0.003	0.331	0.004	−0.006	0.014	0.005	0.449
publication status	−0.057	−0.153	0.040	0.048	0.245	−0.043	−0.184	0.098	0.069	0.539
block 2	*k* = 47; *R*^2^ = 33.65; χ²(2)=3.800; *p* = 0.150					*k* = 31; *R*^2^ = 61.61; χ²(2)=1.579; *p* = 0.454				
publication year	−0.003	−0.008	0.003	0.003	0.321	0.004	−0.006	0.014	0.005	0.434
publication status	−0.055	−0.151	0.041	0.048	0.255	−0.035	−0.180	0.110	0.070	0.624
male ratio	−0.032	−0.153	0.089	0.060	0.602	−0.074	−0.322	0.173	0.120	0.542
mean age	−0.002	−0.004	≥0.001	0.001	0.031	0.001	−0.002	0.005	0.002	0.510
block 3	*k* = 47; *R*^2^ = 26.54; *χ*^2^(4) =7.162; *p* = 0.128					*k* = 31; *R*^2^ = 99.86; *χ*^2^(4) = 14.259; *p* = 0.007				
publication year	−0.001	−0.007	0.005	0.003	0.665	<0.001	−0.009	0.010	0.005	0.937
publication status	−0.030	−0.141	0.080	0.055	0.581	0.093	−0.058	0.243	0.073	0.216
male ratio	−0.038	−0.156	0.081	0.059	0.524	−0.080	−0.289	0.129	0.101	0.438
mean age	−0.001	−0.003	0.001	0.001	0.480	−0.001	−0.004	0.003	0.002	0.745
study goal	−0.076	−0.174	0.023	0.049	0.128	−0.111	−0.265	0.044	0.075	0.152
NoC	−0.016	−0.049	0.018	0.017	0.359	0.086	0.026	0.145	0.029	0.006

Effects on full-scale IQ in patient samples were on the whole weaker and showed a single nominally significant effect of male ratio in block three, indicating smaller associations in men than in women (top right of table 4). However, this finding should be interpreted with caution, because male ratio did not emerge as a significant predictor in any other of our analyses and may be driven by untypically small full-scale IQ correlations of exclusively male patient samples, as evident from our subgroup estimates (table 3).

For verbal IQ, publication year once more negatively predicted effect sizes, but was only significant for healthy samples. In healthy samples, model fit significantly improved when number of corrections within primary studies was included, indicating larger associations when fewer corrections were used. In patient samples, no included predictor yielded significant associations in any block of the model (centre rows of table 4).

For performance IQ, neither variables in the first nor in the subsequent blocks showed significant influences on effect sizes in healthy samples. A similar pattern emerged for patient samples, showing no significant influences in the initial two blocks. Block 3 showed significantly improved model fit compared to Block 1, which was driven by a positive association between the number of corrections within primary studies, indicating smaller associations when fewer corrections were used (bottom of table 4).

### 3.3. Dissemination bias

**Figure 4.**
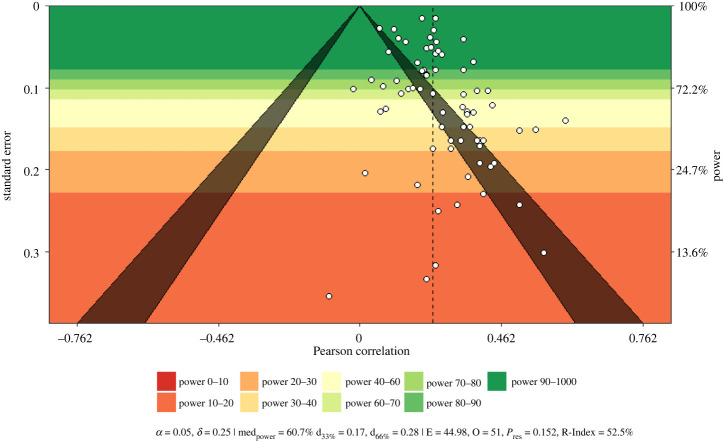
Power-enhanced funnel plot of published healthy sample effect sizes for full-scale IQ.

First, a power-enhanced funnel plot [44] was used to visually inspect full-scale IQ effects of healthy samples. The funnel plot appeared to be fairly symmetrical, but figure 4 shows that most studies had lower power than desirable, yielding a median power estimate of 60.7%. The TES did not provide evidence for confounding dissemination bias (*p* = 0.152), but the replicability index was low (52.5%), thus indicating the presence of inflated results. Median power for verbal and performance IQ was suboptimal as well, yielding 31.2% and 45.9%. Similar to full-scale IQ, TES-results did not indicate dissemination bias in either verbal (*p* = 0.431) or performance IQ (*p* = 0.931), but replicability indices were once again low (13.9% and 38.3%, respectively).

Second, Sterne & Egger's regressions showed significant evidence for funnel-plot asymmetry in full-scale IQ (*p* < 0.001), thus pointing toward confounding dissemination bias. Although there was no bias evidence for verbal IQ (*p* = 0.265), results for performance IQ were once again significant (*p* = 0.011).

Third, trim-and-fill analyses for full-scale IQ indicated 20 missing studies on the left side of the summary effect, leading to an adjusted effect estimate of *r* = 0.21. Although this adjusted estimate should not be used as a correction of the observed summary effect, it serves as a sensitivity analysis that is presently indicative of summary effect inflation. Similar results were observed for verbal and performance IQ were 7 and 11 effect sizes were detected to be missing on the left-hand side, thus yielding adjusted effects of 0.16 and 0.17, respectively (see electronic supplementary material, S1).

Fourth, *p*-curve did not indicate any evidence for confounding *p*-hacking (no evidence for right skew in binomial or continuous tests for full and half *p*-curves; all *p*s < 0.001), and primary studies were on average sufficiently powered to indicate evidential value of the observed non-null effect (all *p*s > 0.999 for binomial and continuous tests against 33% power). *p*-curve-based effect estimations yielded a similar effect to the above reported traditional estimate (*r* = 0.24). Results from *p*-uniform analyses did not indicate confounding bias either (*p* = 0.975), but yielded a somewhat larger summary effect (*r* = 0.26; 95% *CI* [0.22; 0.28]). The estimate based on *p*-uniform* showed a somewhat smaller summary effect than the other two *p-*value-based estimation methods (*r* = 0.22; 95% *CI* [0.18; 0.26]).

Both verbal and performance IQ showed no evidence for *p*-hacking (all *p*s < 0.001), and the available primary studies appeared to provide evidential value (all *p*s > 0.748). Effect estimates were similar to traditional calculations yielding *r* = 0.21 and 0.24, respectively. Results from *p*-uniform did not indicate bias in either domain (*p*s > 0.768) and effect estimates were similar to *p*-curve-based values for verbal (*r* = 0.22; 95% *CI* [0.15; 0.29]) and performance IQ (*r* = 0.23; 95% *CI* [0.16; 0.31]). Once more, *p*-uniform*-based estimates were lower than those of *p*-uniform yielding *r* = 0.18 (95% *CI* [0.11; 0.25]) for verbal and *r* = 0.23 (95% *CI* [0.15; 0.29]) for performance IQ.

Fifth, full-scale IQ-adjusted parameters (i.e. based on the four different weight functions from [200]) did not vary considerably (ranging from *r* = 0.23 to 0.25) and were broadly consistent with the observed unadjusted *r* = 0.25 in.this subset. Results for verbal and performance IQ conformed to this observation, showing rather small variations between different weight function estimates (*r* range for verbal IQ: 0.15–0.19, *r* range for performance IQ: 0.20–0.22) and corresponding broadly to the unadjusted *r*s = 0.20 and 0.23.

By contrast, the selection model of Copas & Shi [54] provided some evidence for dissemination bias in full-scale IQ, indicating 44 missing studies on the left side of the observed summary effect and suggesting an adjusted summary effect of *r* = 0.20. No missing effect sizes were detected for verbal IQ, leading to a virtually identical summary effect of *r* = 0.20, but 11 effects were estimated to be missing on the left side of the performance IQ summary effect, thus leading to an adjustment to *r* = 0.18.

Sixth, confidence intervals estimates for full-scale IQ of the Henmi & Copas [57] approach differed somewhat from the conventional DerSimonian-Laird estimation (95% *CI*s [0.17; 0.27] versus [0.22; 0.28]). For verbal and performance IQ, confidence intervals showed similar patterns (95% *CI*s [0.10; 0.23] versus [0.14; 0.26] and [0.14; 0.26] versus [0.18; 0.28], for conventional and Henmi-Copas estimates, respectively).

Seventh, in a subgroup analysis published summary effects did not differ significantly from unpublished summary effects (i.e. effect sizes from grey literature or personal communications) for full-scale IQ (*Q*(1) = 1.720; *p* = 0.190). However, as expected, published effects were larger than unpublished ones (*r*s = 0.25 versus 0.21, respectively). Results for verbal and performance IQ domains were virtually identical, yielding no nominally significant differences, but consistently larger published summary effects (table 3 for parameters).

**Figure 5.**
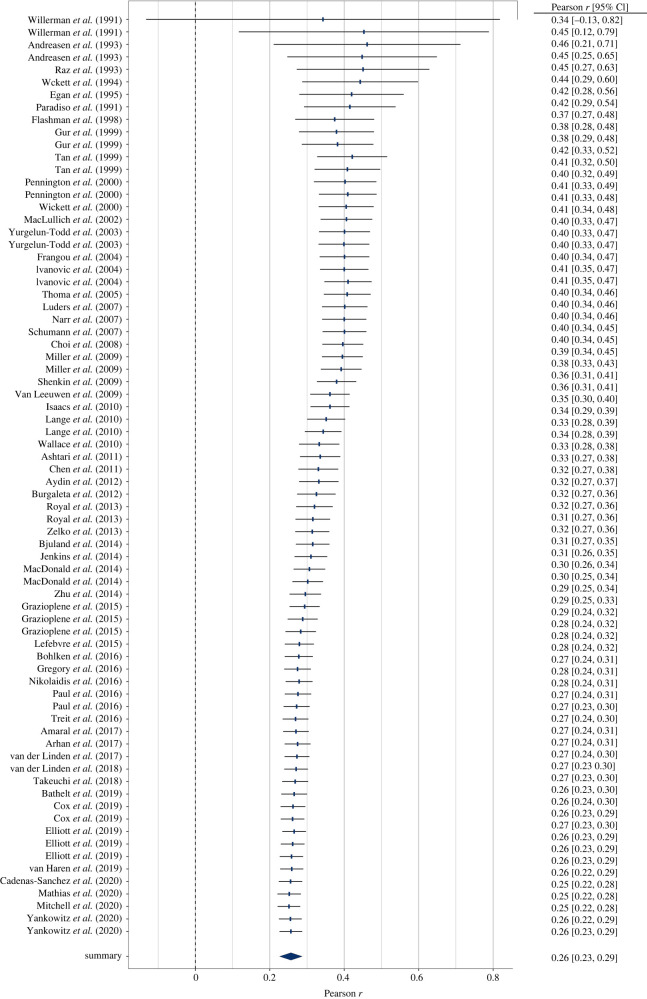
Cumulative forest plot of healthy sample effects according to publication year for full-scale IQ.

**Figure 6.**
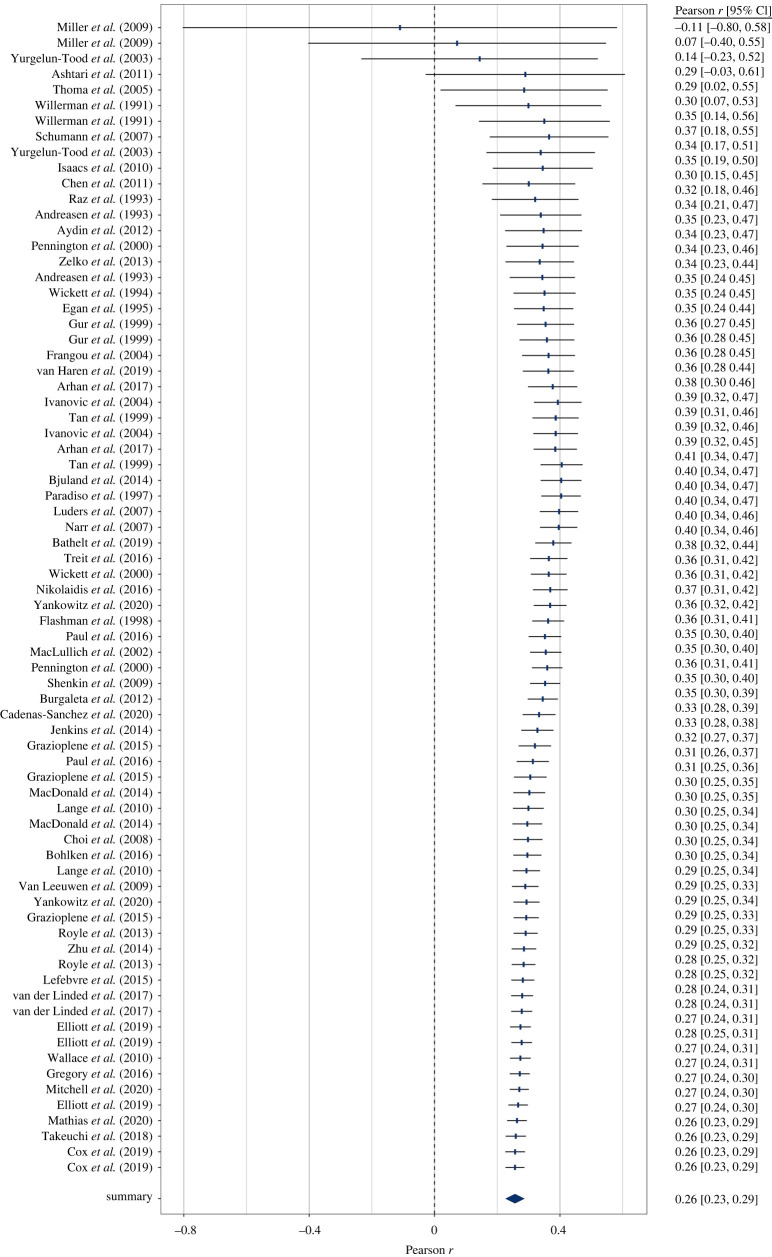
Cumulative forest plot of healthy sample effects according to sample size for full-scale IQ.

Finally, visual inspections of cumulative forest plots of full-scale IQ associations show a clear pattern of systematically decreasing effect sizes over time, thus conforming to our findings of significant negative influences of study years on effect sizes (figure 5). When cumulating results according to sample sizes, a less unequivocal picture emerged, although larger samples appeared to show weaker effects than smaller samples, thus conforming to the above evidence that suggests the presence of some confounding bias in the present data (figure 6). Virtual identical pictures for both publication years and sample sizes result from cumulating verbal and performance IQ data (see electronic supplementary material, S1).

### 3.4. Multiverse meta-analyses

#### 3.4.1. Combinatorial meta-analyses

**Figure 7.**
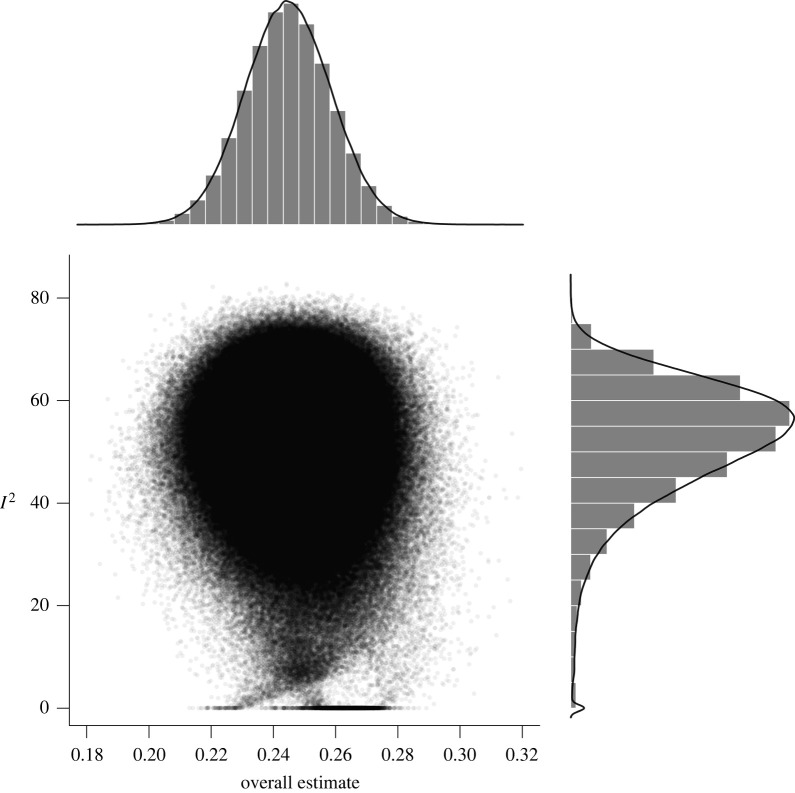
GOSH-plot of 100 000 randomly sampled healthy subsets of all possible combinations for full-scale IQ.

**Figure 8.**
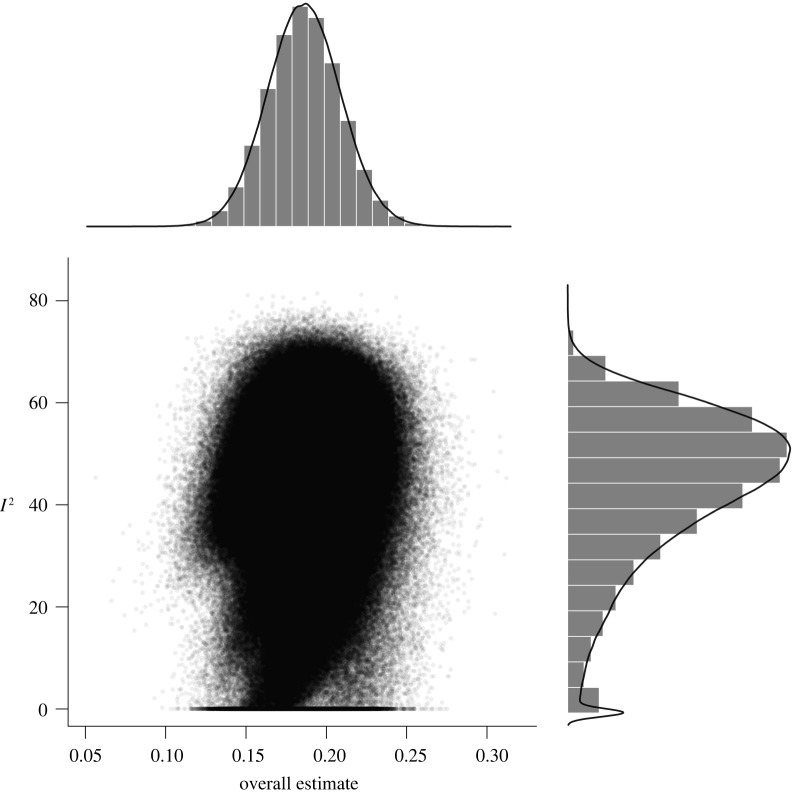
GOSH-plot of 100 000 randomly sampled healthy subsets of all possible combinations for verbal IQ.

**Figure 9.**
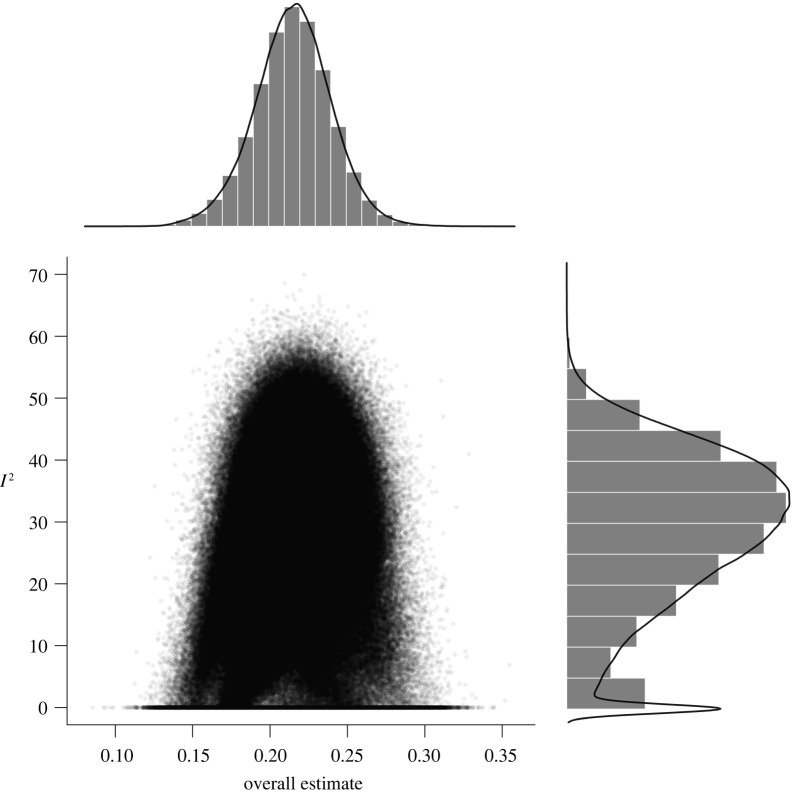
GOSH-plot of 100 000 randomly sampled healthy subsets of all possible combinations for performance IQ.

Results of combinatorial meta-analyses did not show evidence for substantial deviations of the majority of possible combinations from the estimated summary effects. For healthy samples, the interquartile range of the summary-effect distribution for full-scale IQ analyses merely amounted to a difference of 0.02 in *r* values (Q1 = 0.24; Q3 = 0.26; figure 7). Visual inspection of GOSH-plots for the verbal and performance IQ analyses did not indicate systematic patterns either, although interquartile ranges were somewhat larger than for full-scale IQ, most likely owing to the smaller number of included effect sizes (interquartile ranges = 0.03 and 0.03; Q1 = 0.17 and 0.20; Q3 = 0.20 and 0.23; figures 8 and 9). Results of combinatorial meta-analyses for patient samples showed similar patterns and are included in electronic supplementary material, S1.

#### 3.4.2. Specification-curve meta-analyses

**Figure 10.**
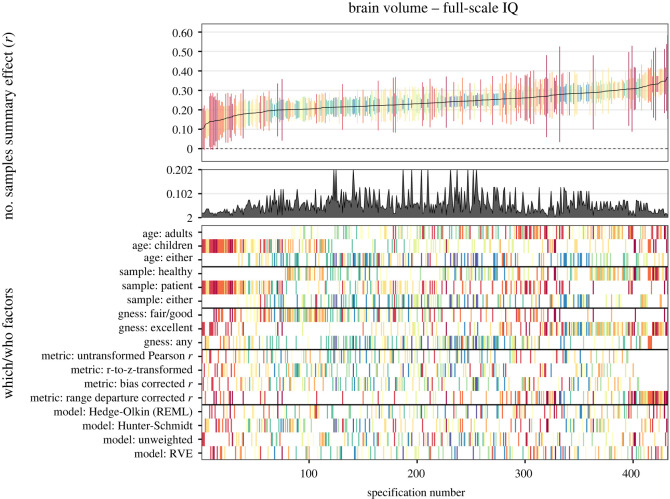
Descriptive meta-analytic specification-curve plot (see [14]) of summary effects from all reasonable specifications for full-scale IQ. Note. The top panel shows summary effects with 95% confidence intervals according to effect strength. The middle panel indicates the number of samples within respective subsets. The bottom panel indicates the respective ‘which’ and ‘how’ factors that were used for the respective effect estimation. Warmer colours indicate lower effect precision (i.e. larger confidence intervals).

**Figure 11.**
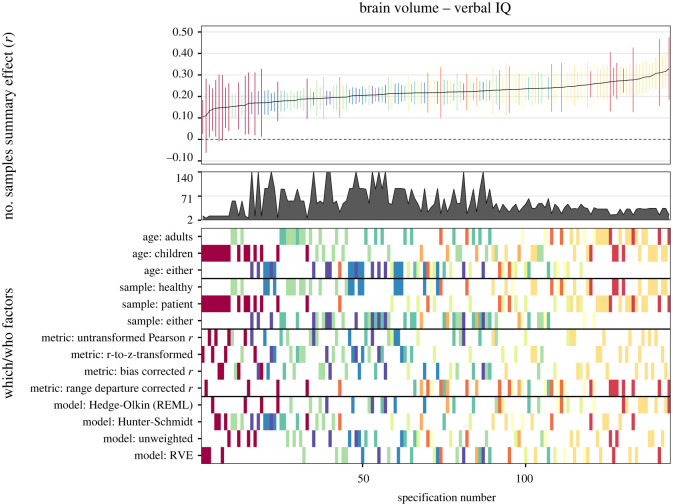
Descriptive meta-analytic specification-curve plot (see [14]) of summary effects from all reasonable specifications for verbal IQ. Note. The top panel shows summary effects with 95% confidence intervals according to effect strength. The middle panel indicates the number of samples within respective subsets. The bottom panel indicates the respective ‘which’ and ‘how’ factors that were used for the respective effect estimation. Warmer colours indicate lower effect precision (i.e. larger confidence intervals).

**Figure 12.**
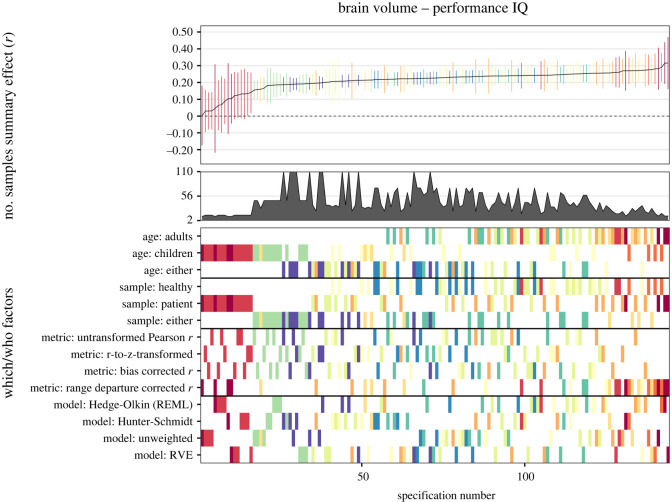
Descriptive meta-analytic specification-curve plot (see [14]) of summary effects from all reasonable specifications for performance IQ. Note. The top panel shows summary effects with 95% confidence intervals according to effect strength. The middle panel indicates the number of samples within respective subsets. The bottom panel indicates the respective ‘which’ and ‘how’ factors that were used for the respective effect estimation. Warmer colours indicate lower effect precision (i.e. larger confidence intervals).

These indicated that virtually any reasonable specification in any domain consistently leads to a positive small-to-moderate association between brain volume and IQ. For full-scale IQ, summary effects ranged from a minimum of *r* = 0.10 to a maximum effect of *r* = 0.37. As can be seen in figure 10, most specifications yielded values in the *r* = 0.20 range. These specifications tended to provide comparatively precise estimates (i.e. effects with narrow confidence intervals), while estimates at the lower and upper end of the effect distribution were less precise. Verbal and performance IQ specification curves were consistent with these observations, almost invariably yielding positive small-to-moderate effects, mostly in the *r* = 0.20 range (figures 11 and 12; minimum *r*s = 0.11 and <0.01; maximum *r*s = 0.33 and 0.32, respectively).

## 4. Discussion

In this quantitative research synthesis, we show that positive associations of *in vivo* brain volume with IQ are highly reproducible. This link is consistently observable regardless of *which* empirical studies are included in a formal meta-analysis and *how* they are analysed. Results of our analyses convergently indicate that the effect strength must be assumed to be small-to-moderate in size, with the best available estimates for healthy participants in full-scale IQ ranging from *r* = 0.24 (uncorrected; approximately 6% explained variance) to 0.29 (corrected approximately 8% explained variance). Effects for full-scale IQ appear to be stronger and more systematically related to moderators compared to verbal and performance IQ. However, these three intelligence domains are highly intercorrelated and their correlation with IQ test results are to be seen as manifestations of a largely similar true effect across domains. We, therefore, focus on full-scale IQ findings of healthy samples in our discussion, unless indicated otherwise.

### 4.1. Comparisons with previous meta-analyses

The strengths of the observed summary effects in the present meta-analysis correspond closely to those identified by Pietschnig *et al*. [24], although the number of participants in this updated analysis is more than three times larger. The observed association for full-scale IQ in healthy samples (i.e. corresponding to selection criteria of the meta-analyses from [25], and [23]) resulted in an estimate of *r* = 0.24 (95% *CI* [0.22; 0.27]), thus indicating considerably lower associations than those reported by Gignac & Bates [25]) and McDaniel [23]). Key characteristics of the available meta-analyses are summarized in table 5.

**Table 5.** Characteristics of available meta-analyses on the *in vivo* brain volume and intelligence link. Note. k = number of independent samples in analysis; summary effect = best estimate according to authors of meta-analysis; when both Hedges & Olkin- as well as Hunter & Schmidt-typed analyses were performed, both estimates are provided, respectively.

	McDaniel [23]	Pietschnig *et al*. [24]	Gignac & Bates [25]	present study
sample type	healthy samples	healthy and patient samples	healthy samples	healthy and patient samples
*K*	37	148	32	194
*N*	1530	8034	2305	26 764
meta-analytic approach	Hunter & Schmidt-typed	Hedges & Olkin-typed	Hunter & Schmidt-typed	Both
summary effect (*r*)	0.33	0.24 (healthy *r* = 0.24; patient *r* = 0.20)	0.40	0.23/0.26 (healthy *r* = 0.24/0.29; patient *r* = 0.21/0.20)
meta-analysis overlap		all studies of McDaniel [23] included	subset of Pietschnig *et al*. [24]	all studies of Pietschnig *et al*. [24] included

It could be argued that these inconsistencies are to a certain extent due to the differing methodological focus of the used analyses because both meta-analyses of Gignac & Bates [25] and McDaniel [23] reported values that were corrected for direct range restriction. However, when we respecified our analyses to apply identical methods, full-scale IQ associations for healthy samples once more led to a lower estimate, yielding *r* = 0.29. This indicates that the reported estimates of prior Hunter & Schmidt-based syntheses were inflated (i.e. even before accounting for dissemination bias).

This idea is supported by our analyses of individual data subsets that used the very same specifications as these prior studies. For instance, Gignac & Bates [25] showed that IQ assessments with higher *g-*ness (i.e. reflecting abilities that are more closely related to psychometric *g*, thus providing a better representation of cognitive abilities) yielded larger associations than less *g*-loaded assessments. They concluded that the most salient estimate of the brain volume and IQ association averages *r* = 0.40 (i.e. corresponding to about 16% of explained variance), based on a specific subset of effect sizes that should provide the most credible results (i.e. using healthy samples, tests with excellent *g-*ness and attenuation-corrected effect sizes only).

None of the reasonable specifications that were included in our specification curve analysis yielded a summary effect that was larger than *r* = 0.37. Importantly, this most extreme upper value of all possible specifications was based on the very same inclusion criteria as the specification that is supposed to represent the best operationalization of this association according to Gignac & Bates [25], healthy samples, excellent *g-*ness, range departure corrected, Hunter & Schmidt estimator), excepting sample age (this uppermost value was based on children/adolescents only; the same specification with all ages yielded *r* = 0.34, corresponding to 11% of explained variance). This is important for a number of reasons.

First, it shows that the specification that was chosen by Gignac & Bates [25] leads to estimates in the extreme upper tail of the distribution of reasonable summary effects. Besides yielding uncharacteristically large values, these estimates have large confidence intervals (i.e. representing higher effect volatility), because they are based on comparatively small sample numbers. Results from our combinatorial meta-analyses showed that at least 75% (i.e. the bottom three quartiles) of results yielded values below *r* = 0.26.

Second, these findings suggest that the estimate reported in Gignac & Bates [25] must be considered to have been inflated, even when one was to assume that this extreme specification yields the most salient estimate for the brain volume and IQ association (i.e. the summary effect in [25], exceeds the upper threshold of any estimate of the present summary effect distribution). Third, the lower summary effects in the present analyses compared to the earlier estimate of Gignac & Bates [25], when identical specifications were used, indicate that the studies that were added in the present update of the literature reported lower correlations, thus conforming to a decline effect [21,22].

Consistent with this interpretation, publication years of primary studies predicted brain volume and IQ associations negatively, indicating decreasing effect sizes over time. Cross-temporally declining effect sizes have been demonstrated to be prevalent in psychological science in general and intelligence research in particular, especially when initial study sample sizes are small [22]. This means that early and small *n* (=imprecise) primary study reports represent more often than not overestimates of the brain size and IQ association, thus having led to inflated meta-analytic summary effects. The presently observed effect declines and comparatively large effect estimates of early small-*n* studies (e.g. [5]) are consistent with the decline effect and its assumed drivers.

### 4.2. Moderators

It is unsurprising that effects were typically stronger in healthy than in patient samples because the included patients suffered from different conditions that are likely to impair cognitive functioning (e.g. autism, brain traumas, schizophrenia) which is bound to introduce statistical noise into the data. Therefore, effects of moderators were substantially weaker and less unequivocal for patients than for healthy samples.

Consistent with Gignac & Bates [25], there were stronger associations with highly *g*-loaded tests compared to fairly *g-*loaded ones in healthy participants (uncorrected *r*s = 0.31 versus 0.19; *Q*(2) = 23.69; *p* < 0.001), but not in patient samples. These results were supported by the findings from our regression analyses where larger *g-*ness positively predicted effect sizes of healthy participants.

Within any examined subgroup, correlations that had been reported within publications were numerically larger than those that had been obtained through personal communications or from the grey literature. This suggests that correlations were selectively reported in the published literature although only differences in full-scale IQ associations of healthy samples reached nominal significance. This observation is consistent with effect inflation because larger associations are more likely than smaller ones to be numerically reported in the literature (numerically stronger effects are more likely to become significant—depending on sample sizes and accuracy—and therefore more likely to be published), thus potentially leading to inadequate assumptions of the readers about the effect strength. This finding is supported by results from our regression analyses that showed weaker effects of unpublished than published effect sizes. This suggests that the reported effects in the brain size and intelligence literature are more often inflated than not, thus conforming to results from Pietschnig *et al*. [24].

In a similar vein, publication years were negatively related to effect sizes, thus indicating a confounding decline effect [21] and conforming to cross-temporally decreasing effect sizes as reported in an earlier meta-analysis [24].

The only further moderator with consistent directions in terms of the observed association appeared to be measurement type which consistently yielded larger estimates for intracranial than for total brain volume, although these differences did not reach nominal significance (except for verbal IQ in patient samples). There were no consistent patterns in regard to age or sex in subgroup or regression analyses, thus conforming to a previous account that indicated that brain volume and IQ associations generalize over participant age bands and sex ([24]; but see [23], for conflicting findings).

### 4.3. Dissemination bias

Three of our formal methods for detecting dissemination yielded significant bias indications for both full-scale and performance IQ (Sterne & Egger's regression, Trim-and-Fill analysis, Copas & Shi's method), while only one method (Trim-and-Fill analysis) indicated bias in verbal IQ. The evidence for bias was stronger for full-scale than performance IQ. It should be noted, that both Sterne and Egger's regression, as well as the Trim-and-Fill analysis, are funnel plot asymmetry-based methods and consequently particularly sensitive for the detection of small-sample effects. This means that the detected bias seems to be rooted in the correspondingly large error variance of underpowered (i.e. small sample size) studies and is consistent with previously raised concerns about suboptimal power in neuroscientific research [201]. Viewed from this perspective, declining effect sizes over time appear to be somewhat reconciliatory, because this may well mean that average study power has increased in this field (or at least in studies addressing this research question).

The low observed replicability indices for all three domains further corroborate the evidence for effect inflation. Similarly, results of our effect estimations by means of *p*-value-based methods support the evidence for confounding dissemination bias, as previously observed in regard to this research question [24]. This interpretation is consistent with larger effects from published sources than from those that were obtained from the grey literature or personal communications, although these differences only reached nominal significance in meta-regressions, but not subgroup analyses.

The present findings contrast the conclusions of Gignac & Bates [25] who did not identify bias evidence in their analysis. This discrepancy may be due to two different causes.

On the one hand, Gignac & Bates [25] included unpublished results in the publication bias detection analyses (i.e. results that [24], had obtained from the grey literature or through personal communications with authors), which (i) prevent potential bias from detection and (ii) are conceptually unsuitable to be used in *p*-curve and *p*-uniform analyses [50,51]. On the other hand, different methods of dissemination bias detection are not equally sensitive for different forms of bias, thus necessitating a triangulation of methods for bias estimation according to current recommendations [42]. Relying on comparatively few and conceptually similar detection methods (i.e. publication bias tests of two *p*-value-based methods; *p*-curve and *p*-uniform; Henmi-Copas approach) may have contributed to the non-detection of bias evidence in this past meta-analysis [25], particularly because these methods are not suitable to detect small-sample effects.

Although the present findings indicate a presence of confounding publication bias, this should not be interpreted as evidence against a brain volume and IQ link. As pointed out above, these associations appear to generalize across numerous potential moderators and replicate well in terms of the identified direction. However, confounding dissemination bias suggests that the obtained summary effects in many primary studies (and even some meta-analyses) represent inflated estimates of the true association. However, it needs to be acknowledged that the future development of more reliable methods for assessing IQ on the one or *in vivo* brain volume on the other hand may lead to larger correlation estimates in primary studies. Nonetheless, the strength of the brain volume and IQ association must be considered to be small-to-medium-sized at best.

### 4.4. Significance of the observed effect

On the one hand, the strength of the observed summary effect suggests that effects of mere neuron numbers, glial cells, or brain reserve are unlikely candidates for the explanation of between-individuals intelligence differences. On the other hand, the effect is clearly non-trivial and has turned out to be remarkably reproducible in terms of its positive direction across a large number of primary studies. Consequently, brain volume should not be seen as a supervenient (i.e. one-to-one) but rather an isomorphic (i.e. many-to-one) proxy of human intelligence. This may mean that brain volume in its own right is too coarse of a measure to reliably predict intelligence differences. It seems likely that examining the role of functional aspects (e.g. white matter integrity) and more fine-grained structural elements (e.g. cortical thickness; see [2]) may help in further clarifying the neurobiological bases of human intelligence.

## 5. Conclusion

In the present meta-analysis, we show evidence for modestly sized associations between *in vivo* brain volume and IQ. The effect appears to be stronger when more *g*-loaded test instruments are used and when full-scale IQ, rather than verbal or performance IQ domains, are assessed. This link appears to be remarkably robust in terms of other potentially moderating variables, generalizing across age, sex or primary study properties, such as study goal and number of variables, that have been controlled for. Importantly, examination of all possible reasonable specifications in regard to how meta-analytical calculations were performed and which studies had been included in the analysis showed consistently positive associations that ranged from *r* = 0.10 to 0.37, with a majority of specifications yielding values around *r* = 0.24. Although in the available literature this link replicated well across studies, some allowance must be made for overestimations of summary effects due to confounding dissemination biases. This indicates that the observed summary effect estimates must be considered to be inflated, thus representing an upper threshold of the true brain size and IQ association.

## Data Availability

All data are available at OSF: https://osf.io/y6msp.
